# Economic evaluation of self-help group interventions for health in LMICs: a scoping review

**DOI:** 10.1093/heapol/czad060

**Published:** 2023-07-27

**Authors:** Jessica Ochalek, Naomi K Gibbs, Rita Faria, Joydeepa Darlong, Karthikeyan Govindasamy, Melissa Harden, Anthony Meka, Dilip Shrestha, Indra Bahadur Napit, Richard J Lilford, Mark Sculpher

**Affiliations:** Centre for Health Economics, University of York, York YO10 5DD, United Kingdom; Centre for Health Economics, University of York, York YO10 5DD, United Kingdom; Centre for Health Economics, University of York, York YO10 5DD, United Kingdom; Research, The Leprosy Mission Trust India, New Delhi 110001, India; Research, The Leprosy Mission Trust India, New Delhi 110001, India; Centre for Reviews and Dissemination, University of York, York YO10 5DD, United Kingdom; Programs Department, RedAid Nigeria, Enugu 400102, Nigeria; Anandaban Hospital, The Leprosy Mission Nepal, Kathmandu Post Box No-151, Nepal; Anandaban Hospital, The Leprosy Mission Nepal, Kathmandu Post Box No-151, Nepal; Institute of Applied Health Research, College of Medical and Dental Sciences, University of Birmingham, Birmingham B15 2TT, United Kingdom; Centre for Health Economics, University of York, York YO10 5DD, United Kingdom

**Keywords:** Cost-effectiveness analysis review, self-help group

## Abstract

This scoping review aims to identify and critically appraise published economic evaluations of self-help group (SHG) interventions in low- and middle-income countries (LMICs) that seek to improve health and potentially also non-health outcomes. Through a systematic search of MEDLINE ALL (Ovid), EMBASE Ovid, PsychINFO, EconLit (Ovid) and Global Index Medicus, we identified studies published between 2014 and 2020 that were based in LMICs, included at least a health outcome, estimated intervention costs and reported the methods used. We critically analysed whether the methods employed can meaningfully inform decisions by ministries of health and other sectors, including donors, regarding whether to fund such interventions, and prioritized the aspects of evaluations that support decision-making and cross-sectoral decision-making especially. Nine studies met our inclusion criteria. Randomized controlled trials were the most commonly used vehicle to collect data and to establish a causal effect across studies. While all studies clearly stated one or more perspectives justifying the costs and effects that are reported, few papers clearly laid out the decision context or the decision maker(s) informed by the study. The latter is required to inform which costs, effects and opportunity costs are relevant to the decision and should be included in the analysis. Costs were typically reported from the provider or health-care sector perspective although other perspectives were also employed. Four papers reported outcomes in terms of a generic measure of health. Contrary to expectation, no studies reported outcomes beyond health. Our findings suggest limitations in the extent to which published studies are able to inform decision makers around the value of implementing SHG interventions in their particular context. Funders can make better informed decisions when evidence is presented using a cross-sectoral framework.

Key messages

- Economic evaluations of self-help group (SHG) interventions in low- and middle-income countries are relatively rare.
- Most studies used randomized controlled trials as the vehicle for data collection and to establish a causal effect, but the costs and outcome measures collected differed between studies.
- Informing decisions around funding SHG interventions with outcomes beyond the health-care sector requires understanding the objectives and values of decision makers in each relevant sector and for the analysis to be able to present conclusions from different perspectives.
- The use of a cross-sectoral framework for reporting the results of economic evaluations can help to ensure that costs, effects and opportunity costs are aggregated appropriately for informing decisions.

## Introduction

Self-help groups (SHGs) are voluntary mutual assistance groups of individuals with a common characteristic, often related to disease status or vulnerability (e.g. leprosy or impoverishment). They exist to meet a collective objective, such as the improvement of health or promotion of well-being (Brody *et al.*, 2015; Gugerty *et al.*, 2019; Martos‐Casado *et al.*, 2020). SHGs have been identified as a promising intervention that can achieve multiple objectives, such as empowerment of members, improved school attendance and health outcomes, while being relatively low cost (Attanasio *et al.*, 2014; Brody *et al.*, 2015; Saha *et al.*, 2015; Aji and Abraham, 2021). Given their potential to affect a range of objectives relevant to health care and other parts of the public sector and wider economy, they may be funded by donors, through government budgets or some combination of funders. Costs may also potentially fall upon individuals in the private sector, such as participants’ time off work to attend SHG meetings and related transportation.

Economic evaluation methods to assess the value of interventions that affect health outcomes to inform resource allocation decisions are well developed (Drummond *et al.*, 2015). Such assessments can provide information to decision makers in the health-care sector (i.e. individuals or organizations such as ministries of health or donors who make decisions around whether to fund an intervention from a given budget over which they have control) on whether the intervention, given the expected costs and health outcomes associated with it and the number of individuals it would be made available to, would be expected to improve health net of any health that will be forgone by others given the finite financial resources available (opportunity cost). Resource allocation decisions that take account of these health opportunity costs ensure decisions improve population health. This is particularly important in low- and middle-income countries (LMICs) where budgets are more restricted; however, a dollar spent on health care is able to purchase more health than in other countries with bigger budgets and better infrastructure (Woods *et al.*, 2016; Ochalek *et al.*, 2018).

Economic evaluation is increasingly being applied in health care in LMICs to inform decisions about which health-care interventions (e.g. treatments and diagnostic tests) should be funded (e.g. through the Ministry of Health’s budget for health care) (Adeagbo *et al.*, 2018; Wilkinson and Chalkidou, 2019; Daccache *et al.*, 2022). However, SHG interventions may reasonably be expected to generate costs and have outcomes outside health care, such as out-of-pocket (OOP) costs to SHG participants and improvements in productivity. While in health care the main objective is generally agreed to be improving population health (Culyer, 2012), the objective of expenditure differs between sectors. Informing decisions on funding SHGs with outcomes beyond the health-care sector requires understanding the objectives and values of decision makers in relevant sectors, data to quantify these outcomes and consensus on how to aggregate the costs and outcomes across different sectors as the basis of decision-making. Recently, attention has been given to how to broaden economic evaluations of potentially health-enhancing interventions when costs and outcomes fall across sectors (Dhaliwal *et al.*, 2013; Walker *et al.*, 2019).

This paper aims to identify and critically appraise the methods used to account for effects and/or costs falling across multiple sectors in evaluations of SHG interventions that seek to improve health, and potentially other outcomes, in LMICs. We consider whether the methods employed can meaningfully inform decisions by ministries of health and other sectors, including donors, regarding whether to fund such interventions, and prioritize the aspects of evaluations that support decision-making and cross-sectoral decision-making especially.

## Methods

We undertook a scoping review of methods proposed or used in evaluations of SHG interventions in LMICs based on the scoping review methodology proposed by Arksey and O’Malley (2007) and developed further by Levac *et al.* (2010) and Munn *et al.* (2018). Our scoping review strategy was informed by previous methodological reviews of health-care interventions in LMICs (Owusu-Addo *et al.*, 2018; Murphy *et al.*, 2019; McMeekin *et al.*, 2020). Our definition of SHGs follows that used in studies by Gugerty *et al.* (2019), Brody *et al.* (2015) and Martos‐Casado *et al.* (2020).

### Defining SHG interventions

**Figure 1.**
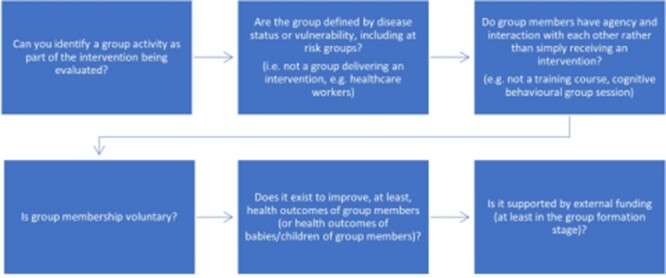
The criteria for the intervention to be classified as including an SHG

SHGs have many and varied definitions in the literature. To ensure consistency and transparency, we produced a set of six conditions drawn from the definitions of self-help used in previous reviews (Figure 1) (Brody *et al.*, 2015; Gugerty *et al.*, 2019; Martos‐Casado *et al.*, 2020) and input from co-authors with expertise around SHGs for leprosy. In brief, we define SHGs as groups supported by external funding that exist to improve the health of members (or of children of members) and potentially also other outcomes, whose membership is voluntary and is defined by disease status or another vulnerability, and where members have agency and interact as part of being in the group. Group members having agency and interacting means they are active participants, as opposed to passive recipients, of an intervention, and mutually assist each other.

#### Search strategy

Our search was designed to identify published economic evaluations of SHG interventions, which included a health outcome, in LMICs. We developed an initial search strategy in Ovid MEDLINE using a range of search terms and subject headings developed by consulting previous reviews (Anderson *et al.*, 2014; Brody *et al.*, 2015; Martos‐Casado *et al.*, 2020), the use of database thesauri and through discussion within the project team. The search terms for SHGs were limited to the more commonly used terms and did not cover all possible alternative terms or synonyms. Given that the purpose of the review was to appraise methods rather than a comprehensive extraction of treatment outcomes, this was deemed appropriate. Terms for SHGs were then combined with a search filter to limit to LMIC countries. A study design search filter was also applied to limit retrieval to economic evaluations (Cochrane Effective Practice and Organisation of Care Group, 2020; Centre for Reviews and Dissemination, n.d.).

We validated our search strategy by checking that it retrieved the economic evaluations relating to health, available in Ovid MEDLINE, which were included in the review conducted by Gugerty *et al.* (2019). As the review by Gugerty *et al.* (2019) covered the literature in this area up to 2014, a date limit of 2014 onwards was applied to the search. No language restrictions were applied to the search.

The final MEDLINE search strategy was adapted for use in all other databases. The following databases were searched to capture studies likely to include health outcomes: MEDLINE ALL (Ovid), EMBASE (Ovid), PsycINFO (Ovid), EconLit (Ovid) and Global Index Medicus, which includes regional databases. All databases were searched on 28 April 2021. The search strategies (including a full list of the regional databases included) can be found in the Supplementary material.

#### Study selection

Our screening strategy aimed to reduce screening time while also ensuring accuracy. All title screening was completed by R.F. J.O. screened an initial 10% of the sample. R.F. and J.O. met to discuss discrepancies. J.O. then screened an additional 10%, and R.F. and J.O. met again to discuss and agree on any discrepancies. All abstracts were screened by both J.O. and N.K.G. Full-text screening was completed by N.K.G. with a 20% sample checked by J.O. Discrepancies at the abstract or full-text screen stage, which remained following discussion, were arbitrated by R.F. and M.S. in the first instance and referred to all co-authors where appropriate.

#### Study inclusion and exclusion criteria

Our inclusion criteria are listed in Table 1. Studies were excluded where they did not meet our definition of self-help, did not include health outcomes and were not quantitative evaluations of an intervention and where the costs included made no attempt to be comprehensive (e.g. the only cost reported was a loan) or they were not in English. Studies were excluded during the title screen only if they were in a high-income country, not a peer-reviewed full-text screen, not in English or clearly unrelated to SHGs. They were excluded during the abstract screen for the same reasons or if the abstract did not include either costs or health outcomes, in methods, results or conclusion, qualitatively or quantitatively; the study design was not an evaluation of an intervention (i.e. quantitative, causal effect, costs and effects); the study was a protocol or the study was a review (systematic or otherwise). If it was unclear from the title and abstract whether the intervention evaluated by the paper was an SHG and all other inclusion criteria were met, then the paper was included in the full-text screen.

**Table 1.** The inclusion criteria

Element	Inclusion criteria
Type of study	Peer-reviewed full-text study, in English
Design	Methodological and/or empirical study Cost-effectiveness, cost-utility, cost–benefit, return on investment, social return on investment, cost-consequence and cost-minimization
Setting	LMIC as defined by the World Bank classification
Intervention	SHG intervention (defined in detail later)
Outcomes	Includes the effect of self-help intervention on, at least, one health outcome Includes the effect of self-help intervention on (at least one) cost aiming to inform a decision
Reporting	Reports methods Reports effect on health outcomes, with or without other outcomes Reports at least one cost

Studies were excluded during the abstract screen using the same criteria applied during the title screen, plus the following criteria: the abstract does not include either costs or health outcomes, in methods, results or conclusion, qualitatively or quantitatively; the study design is not an evaluation of an intervention (i.e. quantitative, causal effect, costs and effects); the study is a protocol and the study is a review (systematic or otherwise). If the intervention evaluated by the paper was not sufficiently explained to be excluded then it was left in during the abstract screen. This meant interventions described only as community mobilization, group support, peer-led, peer-group, participatory learning and action, and microfinance were left in as further clarity needed from the full-text review to ascertain whether these met our definition of SHGs.

#### Critical appraisal

To inform the critical appraisal and to identify best practices and common challenges in accounting for effects and/or costs falling across multiple sectors in evaluations, we developed a data charting form grounded first in the guidelines for a critical assessment of economic evaluation outlined by Drummond *et al.* (2015). In addition to standard bibliographic information, we extracted data on the following study characteristics which are of particular relevance where studies may pertain to multiple sectors: the perspective of the study (i.e. the sector(s) to which costs and outcomes occur and the corresponding decision-making viewpoint(s)), SHG intervention(s) and comparator(s), evidence (including study design, outcome and cost data), value for money and opportunity cost, uncertainty and equity. We gathered additional details to inform whether all relevant costs and outcomes were included, noting which costs and outcomes were included, to which perspective they were applied and the decision (if any) the study aimed to inform. The data charting form was piloted with one cost-effectiveness study (Colbourn *et al.*, 2015) before extracting all studies.

A narrative synthesis of results was undertaken, considering for each study the stated objective, decision maker (stated, implied or neither), the decision (stated, implied or neither), intervention(s) and comparator(s), all reported costs and outcomes and how these were attributed (e.g. to a stated perspective or to inform a stated decision) and how (and whether) opportunity costs were accounted for. We also assessed the time horizon used, how causality is established, whether and how uncertainty is assessed and whether and how equity is considered.

## Results

**Figure 2.**
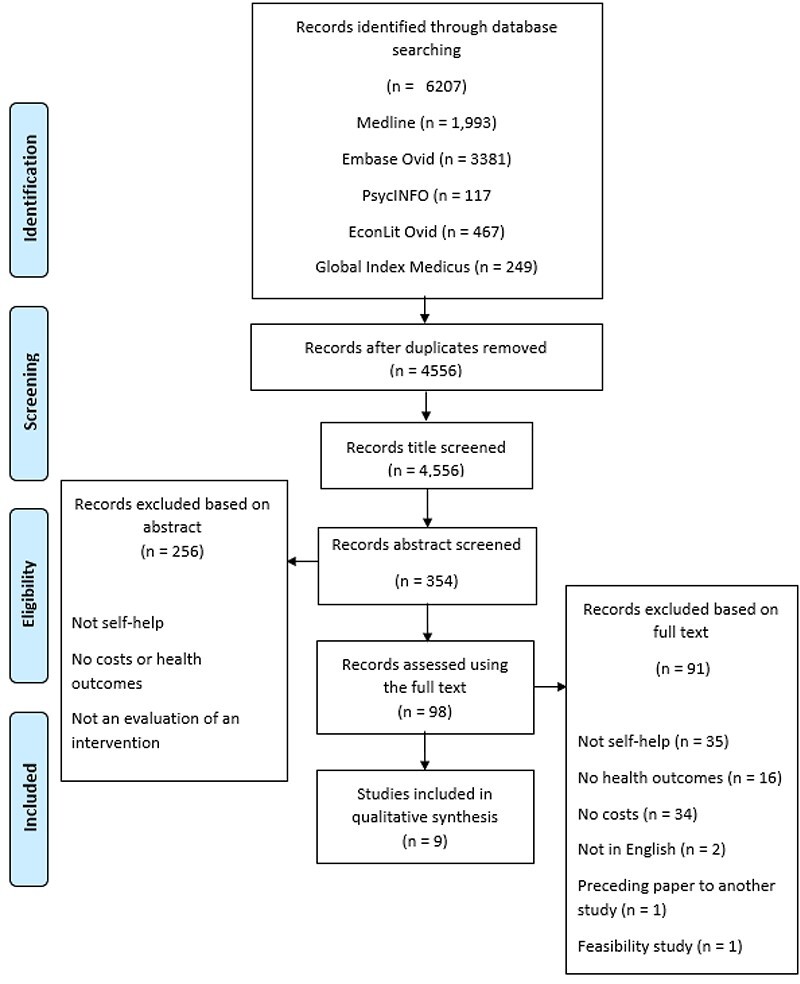
A Preferred Reporting Items for Systematic Reviews and Meta-Analyses diagram

The literature search yielded a total of 4556 records after deduplication, of which 98 studies were full-text screened for eligibility. A total of nine studies met our inclusion criteria and were included in the final review (Figure 2).

### Summary of included studies

Synopses of the interventions and comparators are included in Table 2. As per the inclusion criteria, each study includes the effect of an SHG intervention on, at least, one health outcome. Three of the nine studies are concerned with preventing neonatal mortality (Colbourn *et al.*, 2015; Sinha *et al.*, 2017; Chandrashekar *et al.*, 2019) [of which one also looked at maternal mortality (Colbourn *et al.*, 2015), two were concerned with Type 2 diabetes (Fottrell *et al.*, 2019; Sathish *et al.*, 2020), two with human immunodeficiency virus (HIV)/acquired immune deficiency syndrome (AIDS) (Nichols *et al.*, 2021a,b) and one with intimate partner violence (IPV) (Leight *et al.*, 2021)].

**Table 2.** A summary of included studies

Author(s) (year)	Colbourn *et al.* (2015)	Sathish *et al.* (2020)	Fottrell *et al.* (2019)	Sinha *et al.* (2017)	Chandrashekar *et al.* (2019)	Leight *et al.* (2021)	Oti *et al.* (2016)	Nichols *et al.* (2021a)	Nichols *et al.* (2021b)
Location	Malawi	India	Bangladesh	India	India	Ethiopia	Kenya	Zambia	Lesotho
Target population	Mothers and babies at high risk of mortality	30–60-year olds at high risk of diabetes (according to their Indian Diabetes Risk Score)	Adults >30 years	Women	Women	Adults	Adults 35+	Adults with HIV/AIDS on ART	Adults with HIV/AIDS on ART
Health condition	Maternal mortality and neonatal mortality	Type 2 diabetes	Type 2 diabetes	Neonatal mortality	Neonatal mortality	IPV	Cardiovascular disease	HIV/AIDS	HIV/AIDS
Intervention(s)	1. Community mobilization intervention (CI): participatory learning and action (PLA) women’s groups to identify and prioritize maternal and neonatal health problems, decide upon locally appropriate solutions, advocate for, implement and evaluate the solutions2. Health facility quality improvement intervention (FI): setting up quality improvement teams at health centres3. CI plus FI	1. Fifteen group sessions delivered by trained peer mentors identified from within the group over 12 months focused on behaviours to reduce diabetes risk, and two half-day sessions delivered by experts focused on prevention and management strategies for diabetes	1. Participatory community mobilization: monthly group meetings, led by lay facilitators, applying a PLA cycle focused on diabetes prevention and control 2. mHealth mobile phone messaging: twice-weekly voice messages over 14 months promoting behaviour change to reduce diabetes risk	1. PLA women’s group facilitated by Accredited Social Health Activists aiming to improve maternal and neonatal health through learning, planning, carrying out and evaluating activities in a participatory and sustained basis	1. ‘Layering’ health behaviour change communication focused on reproductive, maternal and newborn health onto existing women’s SHGs with a microfinance component. Where such SHGs did not already exist, the intervention also included forming them	1. Unite for a Better Life groups, which are 20-person men’s, women’s and couple’s groups meeting biweekly for a coffee ceremony (a culturally established forum for community discussion), discussion and interactive activities (with all materials conveyed orally or visually for those not literate) focused on gender norms, sexuality, communication and conflict resolution, HIV/AIDS and IPV to build skills for healthy, non-violent and equitable relationships	1. Support groups and SMS appointment reminders for hypertension patients identified via door-to-door awareness raising and screening household visits by community health workers to promote retention in care. Support groups were incentivized with a group reduction in the price of medication by one-third for collectively achieving 80%+ attendance to follow-up visits for 6 months. Community health workers were financially incentivized to refer screened individuals to clinics and to organize the support groups	Differentiated service delivery models: 1. Community adherence groups: a group of approximately six members, one member collects medication at clinical appointments for other members in a rotating fashion, members meet monthly; 2. Mobile antiretroviral (ART) services; 3. Urban adherence groups: a group of 20–30 patients. Two- to three-monthly group meetings at health facility with adherence counselling by a lay health-care worker followed by prepacked ART dispensation; 4. Home ART delivery	Differentiated service delivery models: 1. Community adherence groups: 6–12 participants. One member collects 3 months’ worth of medication at clinical appointments for other members in a rotating fashion, members meet on a three-monthly basis and all members visit the clinic annually; 2. Community ART distribution: each patient receives one 6-month supply of ART at a clinic visit and another in the community
Comparator(s)	1. Current practice (not reported)	1. Delivery of a health education booklet on standard advice about lifestyle change	1. Current practice, care seeking in government or private facilities, which may involve OOP payment for blood glucose testing, consultations and treatments, and little or no preventative public health campaigning	1. Current practice and no women’s groups. Both trial arms receive monthly meetings with the village health and sanitation and nutrition committees about rights and entitlements	1. Current practice, women’s SHGs with a microfinance-only agenda	1. Short educational 1-h session focused on IPV	Not reported	1. Current practice, clinical visits and ART dispensation at health facility (3-monthly for stable patients and monthly for non-stable patients)	1. Current practice and 3-month, facility-based refills (3MF arm)

The studies were all set within either the Indian subcontinent or sub-Saharan Africa, with no studies being set in the rest of Asia, anywhere in the Americas or North Africa. India was the most common location with three studies (Sinha *et al.*, 2017; Chandrashekar *et al.*, 2019; Sathish *et al.*, 2020). The remaining six studies were in Bangladesh (Fottrell *et al.*, 2019), Malawi (Colbourn *et al.*, 2015), Ethiopia (Leight *et al.*, 2021), Kenya (Oti *et al.*, 2016), Zambia (Nichols *et al.*, 2021a) and Lesotho (Nichols *et al.*, 2021b).

None of the studies qualitatively or quantitatively assess the equity impact of the intervention(s) being evaluated.

### Intervention(s)

Two of the three interventions aimed at reducing neonatal mortality through the implementation of a participatory learning action cycle (PLAC) in a group setting (Colbourn *et al.*, 2015; Sinha *et al.*, 2017). A PLAC broadly involves groups collectively identifying problems around a specific topic (neonatal mortality in this case) and potential strategies for addressing them, actioning some of the strategies and then evaluating the process with a view to improving future iterations. The other intervention (Chandrashekar *et al.*, 2019) aimed at reducing neonatal mortality also involved participatory learning in a group setting. This intervention comprised a health promotion component ‘layered’ onto an existing microfinance SHG, without all the components of a PLAC (as described earlier). The intervention aimed to promote specific behaviours and improve access to health-care services through the facilitation of linkages with front-line health-care workers.

One of the diabetes studies evaluated a PLAC intervention focused on diabetes prevention and control alongside a mobile messaging intervention (Fottrell *et al.*, 2019). The other diabetes intervention (Sathish *et al.*, 2020) was group participatory learning led by trained peer mentors around behaviours to prevent diabetes. The intervention aimed at preventing IPV also involved group participatory learning led by a trained facilitator (Leight *et al.*, 2021).

The SHG component of the other three interventions involved peer support for treatment adherence. In the cardiovascular disease prevention intervention (Oti *et al.*, 2016), support groups were formed to promote retention in care among hypertensive patients who were identified and linked to care in earlier stages of the intervention. The peer support element involved a financial incentive (group reduction in the price of medication) for collective attendance at clinics. Both studies focused on HIV/AIDS, assessed differentiated service delivery models for antiretroviral therapy and included adherence groups as an intervention. Community adherence groups met monthly, with one member collecting medication for other members in a rotating fashion (Nichols *et al.*, 2021a,b). One study (Nichols *et al.*, 2021a) also included urban adherence groups (a type of community adherence group) as an intervention, which involved two- to three-monthly group meetings at health facilities with adherence counselling and ART dispensation.

### Comparator(s)

Six of the nine studies compared the intervention(s) against current practice (Colbourn *et al.*, 2015; Sinha *et al.*, 2017; Chandrashekar *et al.*, 2019; Fottrell *et al.*, 2019; Nichols *et al.*, 2021a,b), and all but one (Colbourn *et al.*, 2015) defined current practice. In one study, current practice is the absence of an intervention (Sinha *et al.*, 2017). In the remaining four studies, current practice refers to existing interventions (Chandrashekar *et al.*, 2019; Fottrell *et al.*, 2019; Nichols *et al.*, 2021a,b). Two papers compare against alternative activities (Sathish *et al.*, 2020; Leight *et al.*, 2021). One paper (Oti *et al.*, 2016) does not specify a comparator.

### Decision context

Three of the nine papers evaluated explicitly state the decision maker that the results of the study would be informative to (Colbourn *et al.*, 2015; Sinha *et al.*, 2017; Chandrashekar *et al.*, 2019). Colbourn *et al.* (2015) note in the introduction that the intervention is funded through donors, who contribute a large proportion of the overall health expenditure in the country, but that future scale-up of the intervention would be expected to be funded by the Ministry of Health. Sinha *et al.* (2017) note that the intervention might be replicated and provided in other parts of India through government systems. Chandrashekar *et al.* (2019) mention that the national government could make a decision to provide the program following the lead of the regional government.

Where papers have not explicitly stated a decision maker, one may be implied via the perspective taken and any information provided about the objective of the paper and who the funder is. For example, in one paper (Sathish *et al.*, 2020) the intervention is funded by a high-income country–based funding body, and the stated objective is to inform the decision to fund the intervention in LMICs, and specifically India, noting the potential for economic evaluation to underpin ‘more effective allocation of scarce healthcare resources … given the country’s ongoing efforts to establish evidence-based health technology assessment to ensure value for money in the health budget’. This paper states that it takes both a ‘health-care system’ (i.e. provider) and societal perspective, and given the objective of ensuring value for money in the health budget, the Ministry of Health can be inferred to be one of the decision makers the paper looks to inform.

### Costs and outcomes

Despite a lack of clarity on average across studies as to the decision context, all studies clearly state a perspective (or perspectives) from which it is decided what costs and outcomes are included. Eight studies state taking a ‘provider perspective’, typically the perspective of the organization providing the intervention. One also states taking a ‘patient’ perspective (Nichols *et al.*, 2021b). The remaining study (Sathish *et al.*, 2020) states taking a ‘health-care system’ and a ‘societal’ perspective.

Similar to the heterogeneity with which the decision context was described across papers, there is also heterogeneity in terms of the costs that the papers report including in their analyses. Four of the studies (Colbourn *et al.*, 2015; Fottrell *et al.*, 2019; Sathish *et al.*, 2020; Nichols *et al.*, 2021b) attribute costs to the Ministry of Health [although in one study (Nichols *et al.*, 2021b), this is only knowable from reading the Supplementary material]. Three studies (Sinha *et al.*, 2017; Chandrashekar *et al.*, 2019; Leight *et al.*, 2021) attribute costs to a donor. Two studies (Oti *et al.*, 2016; Nichols *et al.*, 2021a) do not specify who the provider is.

Costs reported as being included across studies where a provider perspective is taken typically fall under the following categories: health-care utilization, staff, capital, material, programming and medication or lab tests. All papers reported including at least some costs associated with providing the intervention. One paper (Sathish *et al.*, 2020) also attributed private facility expenditure, paid for primarily OOP, to the provider perspective [as recommended by the Second Panel on Cost-Effectiveness in Health and Medicine (Sanders *et al.*, 2016)]. Another (Fottrell *et al.*, 2019) attributed transportation costs, paid OOP by the patient, to the provider perspective. Both these costs would more appropriately be attributed to patients from a societal perspective. Some costs that might reasonably be expected to be included (e.g. staff costs) are not explicitly reported as being included in the studies. It is, however, still possible that they were included, and this underscores the importance of extensive reporting of included costs.

Where a societal or patient perspective is stated, costs reported as being included in the papers are, e.g., private facility or OOP expenditure on health care, expenses incurred while seeking care (e.g. transportation, food and accommodation) and the value of the time lost seeking care. The latter is typically valued in terms of lost wages. Table 3 summarizes the costs reported as included in the analyses of each paper. Cells are highlighted for sectors that costs are attributed to.

**Table 3.** Summary costs reported by papers as being included in the analyses

Author(s) (year)	Colbourn *et al.* (2015)	Sathish *et al.* (2020)	Fottrell *et al.* (2019)	Sinha *et al.* (2017)	Chandrashekar *et al.* (2019)	Leight *et al.* (2021)	Oti *et al.* (2016)	Nichols *et al.* (2021a)	Nichols *et al.* (2021b)
Country	Malawi	India	Bangladesh	India	India	Ethiopia	Kenya	Zambia	Lesotho
Costs attributed to									
Ministry of Health (MoH)	Yes	Yes^a^	Yes^a^^,^^b^	No	No	No	No	No	Yes^b^
Health-care utilization	No	Yes	Yes						Yes
Staff	Yes	Yes	No						Yes
Capital	Yes	No	No						Yes
Material	Yes	Yes	No						Yes
Joint programming costs	Yes	No	No						Yes
Medication and lab tests									Yes
Donor	No	No	No	Yes	Yes	Yes	No	No	No
Health-care utilization				No	No	No			
Staff				Yes	Yes	Yes			
Capital				Yes	No	Yes			
Material				No	Yes	Yes			
Joint programming costs				Yes	Yes	Yes			
Medication and lab tests									
Provider (not specified)	No	No	No	No	No	No	Yes	Yes	No
Health-care utilization							Yes	Yes	
Staff							Yes	Yes	
Capital							Yes	No	
Material							Yes	No	
Joint programming costs							Yes	No	
Medication and lab tests							Yes	Yes	
Society	No	Yes	No	No	No	No	No	No	Yes
Private facility expenditure (paid OOP)		No							No
Expenses for transport/food/accommodation		No	Yes						Yes
Time lost from seeking care		No							Yes

a Some costs falling in other sectors are attributed to this sector.

b The paper specifies the provider perspective but does not state which sector the provider perspective refers to, one must be inferred.

Only four of the nine papers reported the effect of the intervention on costs related to the use of health-care services. For example, one paper (Fottrell *et al.*, 2019) estimated savings in health-care costs from the reduced use of health-care services by intervention recipients.

The outcome measures used across the papers varied and are summarized in Table 4. None of the studies report outcomes beyond health, such as well-being or microfinance outcomes. Four papers (Colbourn *et al.*, 2015; Sinha *et al.*, 2017; Fottrell *et al.*, 2019; Sathish *et al.*, 2020) reported a final generic health outcome measure [i.e. quality-adjusted life years (QALYs) or disability-adjusted life years (DALYs)]. All four also reported a final survival health outcome measure (e.g. mortality or life expectancy). One paper only reported a final survival health outcome measure (Sinha *et al.*, 2017). Two papers only reported intermediate health outcomes (Oti *et al.*, 2016; Leight *et al.*, 2021) (e.g. cases detected, cases averted and patient with blood pressure controlled). Another two only reported process outcomes (i.e. patient retained in care at 12 months) (Nichols *et al.*, 2021a,b). One study reported all four types of outcome measures (Fottrell *et al.*, 2019).

**Table 4.** A summary of outcome measure(s) reported

Author(s) (year)	Colbourn *et al.* (2015)	Sathish *et al.* (2020)	Fottrell *et al.* (2019)	Sinha *et al.* (2017)	Chandrashekar *et al.* (2019)	Leight *et al.* (2021)	Oti *et al.* (2016)	Nichols *et al.* (2021a)	Nichols *et al.* (2021b)
Country	Malawi	India	Bangladesh	India	India	Ethiopia	Kenya	Zambia	Lesotho
Outcome measure(s) reported	DALYs averted	QALYs, cases detected	Cases averted of diabetes, cases averted of intermediate hyperglycaemia and DALYS	DALYs averted, neonatal death averted	LYs saved	IPV case averted	Patient with blood pressure controlled	Patient retained in care at 12 months	Patient retained in care at 12 months
Type of outcomes									
Final generic outcome measure (DALY and QALY)	Yes	Yes	Yes	Yes	No	No	No	No	No
Final survival outcome measure (e.g. mortality, life expectancy and quality-adjusted LE)	Yes	Yes	Yes	Yes	Yes	No	No	No	No
Intermediate outcomes (e.g. CD4 cell count and incidence of T2DM)	No	Yes	Yes	No	No	Yes	Yes	No	No
Process outcomes (e.g. adherence and physical activity)	No	No	Yes	No	No	No	Yes	Yes	Yes

All three studies that report a DALY outcome measure calculate DALYs averted by combining deaths averted with the standard life expectancy at birth used in the Global Burden of Disease Study (86 years), which is higher than local life expectancy at birth in each of the countries studied(Malawi, Bangladesh and India) (Colbourn *et al.*, 2015; Sinha *et al.*, 2017; Fottrell *et al.*, 2019). One study considers local life expectancy in addition and notes that the cost per DALY averted is higher but does not use this in their main results (Colbourn *et al.*, 2015). None of the three studies adjust survival for the quality of life in which additional years will be lived, implicitly assuming that years of life gained will be lived in perfect health.

### Aggregating costs and outcomes

In order to support decision makers’ assessment of whether an SHG is good value, it is necessary to assess outcomes alongside costs (i.e. to aggregate costs and outcomes). Six of the nine papers reported on whether the intervention was deemed cost-effective or not (Colbourn *et al.*, 2015; Sinha *et al.*, 2017; Chandrashekar *et al.*, 2019; Fottrell *et al.*, 2019; Sathish *et al.*, 2020; Leight *et al.*, 2021) (Table 4). The remaining three reported only intermediate and process outcomes (e.g. patient with blood pressure controlled) (Oti *et al.*, 2016; Nichols *et al.*, 2021a,b). Of the six papers that made judgements about the cost-effectiveness of the interventions, all concluded the intervention being evaluated to be cost-effective by comparing an incremental cost-effectiveness ratio (ICER, which is the ratio of the incremental change in costs against the next best alternative to the incremental change in outcomes against the same) against a cost-effectiveness threshold. Three of the six papers that judged cost-effectiveness based their judgement on the estimated cost per DALY averted being <1× gross domestic product (GDP) per capita (Colbourn *et al.*, 2015; Sinha *et al.*, 2017; Fottrell *et al.*, 2019). One based this on the estimated cost per QALY gained being <3× GDP per capita (Sathish *et al.*, 2020). One based this judgement on the estimated cost per life year (LY) gained being <3× GDP per capita (Chandrashekar *et al.*, 2019). One, noting the inability to convert the estimated outcome into a final generic measure, based their judgement on a comparison to the status quo judging the cost per IPV case averted by the intervention to be comparable to or lower than other IPV prevention interventions (Leight *et al.*, 2021). Results are reported in Table 5.

**Table 5.** A summary of cost-effectiveness judgements made across studies

Author(s) (year)	Colbourn *et al.* (2015)	Sathish *et al.* (2020)	Fottrell *et al.* (2019)	Sinha *et al.* (2017)	Chandrashekar *et al.* (2019)	Leight *et al.* (2021)	Oti *et al.* (2016)	Nichols *et al.* (2021a)	Nichols *et al.* (2021b)
Reported ICER	Yes	Yes	Yes	Yes	Yes	Yes	No	No	No
Reported net benefit	No	No	No	No	No	No	No	No	No
Judged cost-effectiveness	Yes	Yes	Yes	Yes	Yes	Yes	No	No	No
Cost-effectiveness conclusion	Yes	Yes	Yes	Yes	Yes	Yes	Not reported	Not reported	Not reported
Cost-effectiveness judged by	1× GDP per capita	3× GDP per capita	1× GDP per capita	1× GDP per capita	3× GDP per capita	Comparison to the status quo			

### Time horizon

The appropriate time horizon is the full duration of differences in costs or effects existing between those receiving SHG and those not, which is lifetime in any situation where there is an impact on survival. The time horizon used is reported in Table 6. Four studies use a 12-month time horizon (Oti *et al.*, 2016; Leight *et al.*, 2021; Nichols *et al.*, 2021a,b). These are the same four studies that report only process or intermediate outcome measures. Two of the four studies (Nichols *et al.*, 2021a,b) are primarily concerned with estimating cost differences between different models of provision of care. One focuses on describing costs and outcomes rather than assessing value (Oti *et al.*, 2016). The fourth study only examines cases of past-year violence averted (Leight *et al.*, 2021). None of the four studies consider the duration of effect beyond 1 year.

**Table 6.** A summary of time horizon across studies

Author(s) (year)	Colbourn *et al.* (2015)	Sathish *et al.* (2020)	Fottrell *et al.* (2019)	Sinha *et al.* (2017)	Chandrashekar *et al.* (2019)	Leight *et al.* (2021)	Oti *et al.* (2016)	Nichols *et al.* (2021a)	Nichols *et al.* (2021b)
Time horizon	10 years	2 years	Not reported	28 months	Not reported	12 months	12 months	12 months	12 months

Among the remaining five studies, two use time horizons that correspond with the average length of trial follow-up (Sinha *et al.*, 2017; Sathish *et al.*, 2020). One of these studies does not calculate outcomes beyond the trial period; however, the authors acknowledge that the duration of effect is expected to be longer and suggest that the benefits of the intervention may be underestimated as a result of the truncated time horizon (Sathish *et al.*, 2020). The other paper estimates the effect of the SHG intervention on neonatal mortality and calculates DALYs averted over the expected life time (Sinha *et al.*, 2017). This is also done by Fottrell *et al.* (2019) and Chandrashekar *et al.* (2019). None of these estimate costs beyond the duration of the study.

One study uses a 10-year time horizon based on guidance from the World Health Organization (World Health Organization *et al.*, 2003; Colbourn *et al.*, 2015). They extrapolate costs and outcomes measured over the duration of the trial (2.25 years) to 10 years and lifetime, respectively.

### Establishing causality in intervention effects

Eight of the nine studies undertake an empirical evaluation to estimate a causal effect of the intervention, with the majority (seven) being based on randomized controlled trials (RCTs) (Colbourn *et al.*, 2015; Sinha *et al.*, 2017; Fottrell *et al.*, 2019; Sathish *et al.*, 2020; Leight *et al.*, 2021; Nichols *et al.*, 2021a,b). Among these, two used a cluster randomized non-inferiority trial design (Nichols *et al.*, 2021a,b). Each of these studies established a causal effect.

There were two quasi-experimental evaluations (Oti *et al.*, 2016; Chandrashekar *et al.*, 2019). One of these employed both an empirical evaluation and a decision model (Chandrashekar *et al.*, 2019). The authors established a causal effect of the intervention using a difference-in-difference approach. The other study estimated outcomes from before and after the intervention (Oti *et al.*, 2016), and without the comparison group, this cannot be considered robust as there is no way to know how the participants would have fared in the absence of the intervention.

### Accounting for uncertainty

Uncertainty in the evidence from studies may arise from sampling uncertainty (i.e. small samples), uncertainty regarding assumptions about how effects evolve over time (particularly when extrapolating beyond study follow-up) or uncertainty about the validity of identifying assumptions in the empirical analysis. All but one (Oti *et al.*, 2016) of the studies reviewed included some form of sensitivity analysis with most studies having conducted at least one-way sensitivity analysis (Colbourn *et al.*, 2015; Sinha *et al.*, 2017; Chandrashekar *et al.*, 2019; Fottrell *et al.*, 2019; Sathish *et al.*, 2020; Leight *et al.*, 2021; Nichols *et al.*, 2021b). One also conducts a multi-way sensitivity analysis (Sathish *et al.*, 2020). Two studies undertook probabilistic sensitivity analysis (Colbourn *et al.*, 2015; Nichols *et al.*, 2021a). Results are reported in Table 7.

**Table 7.** A summary of how studies account for uncertainty

Author(s) (year)	Colbourn *et al.* (2015)	Sathish *et al.* (2020)	Fottrell *et al.* (2019)	Sinha *et al.* (2017)	Chandrashekar *et al.* (2019)	Leight *et al.* (2021)	Oti *et al.* (2016)	Nichols *et al.* (2021a)	Nichols *et al.* (2021b)
Model or empirical evaluation	Empirical evaluation	Empirical evaluation	Empirical evaluation	Empirical evaluation	Empirical evaluation and model	Empirical evaluation	Neither	Empirical evaluation	Empirical evaluation
Method for establishing a causal effect	Cluster RCT	Cluster RCT	Cluster RCT	Cluster RCT	Quasi-experimental (difference-in-difference), decision tree	Cluster RCT	Before and after comparison	Cluster randomized non-inferiority trial	Cluster randomized non-inferiority trial
Type of uncertainty analysis undertaken	One-way and probabilistic sensitivity analyses	One-way and multi-way sensitivity analyses	One-way sensitivity analysis	One-way sensitivity analysis	One-way sensitivity analysis	One-way sensitivity analysis	Not reported	Probabilistic sensitivity analysis	One-way sensitivity analysis

## Discussion

The aim of economic evaluation is to provide evidence to support decision-making. Crucial to this is who the decision maker is and what the objectives of the sector are. Also crucial are the costs, outcomes associated with the decision, on whom they fall and the resulting opportunity costs. What outcomes matter depends upon the objectives of the sector in which decisions are being made. What these objectives are is a question of social value.

With a view to capturing all relevant outcomes to decision-making across sectors, the Second Panel on Cost-Effectiveness in Health and Medicine recommended presenting outcomes in the form of an ‘impact inventory’ where these are disaggregated by sector, and also reporting a summary measure [e.g. ICER, net monetary benefit or net health benefit (Sanders *et al.*, 2016)]. While reporting in this disaggregated fashion is more transparent and arguably more informative than combining all costs and benefits into their monetary value, it falls short of providing evidence on whether investing in an intervention would generate more value than an alternative use of the same resources (i.e. it also fails to account for the opportunity cost of expenditure). There are three examples of studies in our review that do not aggregate outcomes (e.g. Oti *et al.*, 2016; Nichols *et al.*, 2021a,b; see Table 5), which limits their usefulness for decision-making.

More commonly among the studies assessed here, aggregation takes the form of an ICER. Across the health economics literature, the health outcome denominator in ICERs may be reported in terms of a generic health outcome or narrower outcomes, such as survival outcomes, intermediate or process outcomes. Using a narrow health outcome measure (as in four of the six studies that report ICERs) limits the value of the results of a study to inform resource allocation decisions because of the lack of comparability in such measures. For example, the value of a patient retained in care at 12 months (a process outcome) cannot be directly compared against the value of an IPV case averted (an intermediate outcome) or against a DALY averted (a final generic outcome measure). It is therefore important to be able to convert process, survival and intermediate outcomes into final generic outcome measures. The authors of the paper assessing the IPV prevention intervention raise the lack of existing DALY estimate corresponding to past-year exposure to IPV to justify their inability to provide comparisons of IPV reduction intervention against other interventions to improve health, but this remains an important limitation of the study (Leight *et al.*, 2021).

QALYs and DALYs are the most widely used final generic outcome measures in health, incorporating changes to both the length and quality of life, enabling comparisons of interventions across different disease areas. Fewer than half of the studies assessed as part of this review reported a final generic health outcome measure, the use of which facilitates comparison with the opportunity cost of funding the intervention. Exactly what existing health care is defunded (or what potential health care is not funded from a budget expansion) to fund the intervention cannot typically be known, and so on whom precisely the opportunity costs fall is unknown (Lomas *et al.*, 2021). Estimates are available, however, of the amount of health (in terms of QALYs or DALYs) that would be gained from an increase, or lost from a decrease, in the budget for health care (Woods *et al.* 2016; Ochalek *et al.*, 2018, 2020; Edoka and Stacey, 2020; Espinosa *et al.*, 2022; Moler-Zapata *et al.*, 2022). This enables the cost of the intervention to be expressed as the health that could have been gained with the funding (i.e. its health opportunity cost). The expected health gain from the intervention net of the health opportunity cost is the expected net health benefit, which, unlike ICERs, can reflect the magnitude to which an intervention is more or less cost-effective than an alternative. The merits of the net benefit metric over ICERs have been described in detail elsewhere (Paulden, 2020a,b).

Among the studies that used ICERs to make judgements around the cost-effectiveness of the intervention being analysed, none of these studies used thresholds that reflected the opportunity cost of funding the intervention. All instead employ heuristic ‘rule of thumb’ decision rules. One study (Leight *et al.*, 2021) compares the cost per case of past-year physical and/or sexual IPV averted against other interventions with the same outcome measure. This provides information on the relative cost-effectiveness of this intervention against others with a similar objective but is not able to inform decision makers about the value of providing this intervention against how much health could be generated by the resources required to fund it if spent on other existing health cares within the health-care system. The other five studies apply a GDP-based threshold rule, either 1× or 3× GDP, which were previously recommended by the World Health Organization to categorize interventions as very cost-effective or cost-effective, respectively (Bertram *et al.*, 2016). While widely applied as a cost-effectiveness threshold range, these GDP-based norms do not reflect health opportunity costs. As such, it is possible for an intervention to be considered ‘highly cost-effective’ according to this threshold range, while at the same time, if funded, it would reduce overall population health (Woods *et al.*, 2016; Ochalek *et al.*, 2018).

All of the studies included assess the costs and effects of SHGs on health. However, the objectives of SHGs may be broader, and such groups may have costs and outcomes beyond the health-care sector. For example, an SHG intervention with costs falling on a publicly funded health-care sector and on individuals (e.g. the cost of providing the intervention for the former and the cost of transportation to attend the intervention for the latter) might be expected to have benefits in terms of health and reduced absenteeism at work or school (i.e. benefits accrued to the health-care sector, the labour market and/or the education sector). In this case, decision makers may wish to consider the opportunity cost of expenditure in the health-care sector and in the private sector. These opportunity costs would be expected to differ because of the marginal cost (and value) of public funds, the literature on which suggests that the opportunity cost of expenditure falling on the health-care sector is higher than that falling on the private sector (Walker *et al.*, 2012; Finkelstein and Hendren, 2020). One of the studies reviewed assessed a health-focused intervention that was ‘layered onto’ microfinance SHGs (Chandrashekar *et al.*, 2019). Only two papers (Sathish *et al.*, 2020; Nichols *et al.*, 2021b), however, attempt to account for potential costs or outcomes falling on other sectors. Both consider costs falling on society including, e.g., expenses for transport, food and accommodation when seeking care and time lost from seeking care and Nichols *et al.* (2021b) also consider private facility expenditure paid OOP.

One proposed approach for dealing with costs and outcomes falling outside the health-care sector is for analyses to take a ‘societal perspective’, where all costs and outcomes are included in the analysis regardless of who incurs them (Sanders *et al.*, 2016). Some have attempted to operationalize this by combining costs and benefits falling across different sectors into their monetary value and aggregating (e.g. as in cost–benefit analysis). In health care, e.g., benefits may be monetized using estimates of the consumption value of health (Lomas *et al.*, 2022). However, this approach implicitly assumes that there are no budget constraints on public sectors and therefore fails to account for the opportunity cost of expenditure (Claxton *et al.*, 2010).

One of the studies assessed (Sathish *et al.*, 2020) takes both a provider perspective and a societal perspective. The authors implement the societal perspective by including transportation, food and accommodation costs incurred by patients, and the value of time spent seeking care (measured in terms of lost wages), in addition to the intervention costs they attribute to the health-care system. The authors attribute private facility expenditure, paid for primarily OOP, to the provider perspective. This is consistent with the existing guidance from the Second Panel on Cost-Effectiveness in Health and Medicine (Sanders *et al.*, 2016); however, the opportunity cost of this expenditure falls upon private individuals rather than on the government budget for health care. We, therefore, would argue that such costs would be better separated from the health system and attributed to the individuals participating. The authors apply the same outcome measures under both perspectives, implying that health outcomes represent all important objectives from the intervention (within and beyond the health-care sector). They also apply the same cost-effectiveness threshold to decide whether the intervention is value for money under each perspective. This can be interpreted as treating non-health-care costs (i.e. those falling on patients in this case) as if they fall on the health-care system, which can provide misleading information to decision makers.

Furthermore, interventions may have important outcomes that fall beyond what is deemed relevant to the decision maker a priori. This suggests that as much data as can be collected should, and it is useful to have an understanding of the opportunity cost of an intervention so that decision makers can transparently deliberate about whether these other benefits gained (whether anticipated or beyond what was initially anticipated and deemed relevant) outweigh any perspective-specific benefits forgone as a result of the opportunity cost of funding the intervention. However, collecting additional data adds costs and additional complexity to studies. As such, a priori judgements are required about where differences between interventions are expected. However, decision makers and researchers may not always know this in advance. There may, therefore, be scope for preliminary or pilot studies to inform judgements about the likelihood of there being differences in a range of outcomes and the likelihood that these matter.

The majority (seven) of the studies reviewed here established a causal effect using RCTs. RCTs generally have high internal validity conditional on appropriate implementation to ensure randomization. Non-experimental data analysed using quasi-experimental approaches return a causal estimate only if identifying assumptions are met. In the case of the difference-in-difference approach (employed in Chandrashekar *et al.*, 2019), this is known as the parallel trend assumption, which requires the intervention and non-intervention groups to have the same continuation in trends over time in the absence of the intervention. Data on the same group before and after the intervention provides a weaker form of evidence due to the lack of a control group. Analysis of these data cannot be expected to return a causal estimate except under very strong assumptions.

Informing decisions requires appropriately accounting for uncertainty within the evidence. Uncertainty analysis can inform a decision to implement a policy or wait until further evidence develops and some of the uncertainty is resolved. There are various methods of handling uncertainty with probabilistic sensitivity analysis being the preferred approach as it both allows the uncertainty associated with different parameters to be reflected simultaneously in the model results and provides the best estimates of mean costs and outcomes in non-linear decision models where outputs are a result of a multiplicative function (e.g. in Markov models) (National Institute for Health and Care Excellence, 2012). Two of the studies reviewed conducted a probabilistic sensitivity analysis (Colbourn *et al.*, 2015; Nichols *et al.*, 2021a). One conducted no sensitivity analysis (Oti *et al.* 2016). The remaining studies conducted only one-way sensitivity analyses. One-way sensitivity analysis, while useful, e.g. in model development, is limited as it is unable to represent the combined effect of uncertainty in the value of all input parameters at once provided by probabilistic sensitivity analysis.

Six of the studies reviewed use a time horizon based on the duration of the study follow-up, ranging from 6 to 28 months (Oti *et al.*, 2016; Sinha *et al.*, 2017; Sathish *et al.*, 2020; Leight *et al.*, 2021; Nichols *et al.*, 2021a,b). Any time horizon short of a lifetime assumes that there are no differences in costs and outcomes between people who have had the intervention and those who have not once the time horizon is passed. In some circumstances, this can be valid, although in many cases a bias is introduced where important long-term outcomes are missed. The time horizon used should reflect the full duration of differences in costs or effects between the intervention and non-intervention groups. Where there is an effect on mortality or survival, this would be a lifetime time horizon. Modelling approaches can extrapolate results to a more distant time horizon, but this also introduces additional uncertainty.

Three studies in this review extrapolated outcomes over the lifetime by converting estimated effects on mortality into DALYs averted (Sinha *et al.*, 2017; Chandrashekar *et al.*, 2019; Fottrell *et al.*, 2019). All used, at least as their base case, the maximum achievable life expectancy across the globe, which is higher than local life expectancy in all cases. This results in an overestimation of the effect size. Only one (Fottrell *et al.*, 2019) weighted the years of life gained by the quality in which they were expected to be lived. The remaining two implicitly assumed perfect health by neglecting to apply a quality-of-life weight or decrement to the years of life gained (Sinha *et al.*, 2017; Chandrashekar *et al.*, 2019). This also biases the effect size upwards.

None of the studies reviewed here made an attempt to account for equity within their analyses. Improvement in equity, however that is measured, may be one of the decision maker’s objectives in sectors where SHG outcomes occur, and SHG interventions themselves may be targeted towards helping the poorest in society. One of the SHG interventions included in this review explicitly targeted people living in slums (Oti *et al.*, 2016), while another focused on women vulnerable to IPV [56]. Frameworks are available to incorporate equity concerns. Distributional cost-effectiveness analysis (DCEA) presents the net health effects of an intervention in terms of health outcomes disaggregated by relevant groups within the population, such as socio-economic wealth quintile (Asaria *et al.*, 2015; Cookson *et al.*, 2017). Health opportunity costs are accounted for in the measure of net health effects, requiring data on the distribution of baseline health and health opportunity costs by equity-relevant subgroups. The framework set out by Walker *et al.* (2019) is compatible with DCEA, also enabling the incorporation of equity concerns by sector. Extended cost-effectiveness analysis, although not explicitly requiring the incorporation of opportunity cost, does specify the inclusion of financial risk protection, an outcome often highly relevant in LMIC settings (Verguet and Jamison, 2017).

The challenges to conducting economic evaluation are numerous, and this is particularly true in LMICs where data tend to be sparser. The studies reviewed here all state a perspective (or perspectives) from which it is decided what costs and outcomes are included and typically reported costs and outcomes transparently. This is important to inform the transferability of data between settings (as, e.g., health-care utilization costs may differ). Reporting of costs and outcomes separately also enables the calculation of an intervention’s expected net health benefit in different countries by applying country-specific estimates of the cost per unit of health produced by the health-care system. This would be expected to differ between countries due to differences in health spending, infrastructure for delivering health care, the size and age and gender distribution of the population, as well as the burden of disease in a country (Ochalek *et al.*, 2018). The studies are transparent about their limitations where these exist (e.g. using a process or intermediate outcome measure), making it possible for research users to interpret the evidence and potential biases that may be relevant.

These recommendations apply to many different types of interventions and are not specific to SHG interventions. SHG interventions are fairly unique in health economic evaluation as they more often than not also have other objectives, and most typically, we find, do not include as an objective the improvement of health at all. Economic evaluation of interventions with effects on health and other non-health outcomes remains an area under development. Future studies evaluating SHG interventions should ensure that their design takes direct account of the objectives of the sector(s) and decision maker(s) the study aims to inform. Where an SHG intervention aims to improve health and may be considered for funding as part of a nationally provided health benefits package or other pooled government funds for health care, evaluations should report results in terms of generic health outcome measures (such as QALYs or DALYs) so that the intervention can meaningfully be compared against other health-care interventions, which may be competing for the same funds but targeting different sub-populations and diseases. Where the objectives of the SHG are broader than improvements in health, the objectives of the other relevant sector(s) should be accounted for in the analysis. For example, if one of the objectives of an SHG is to increase consumption, the objectives of the Ministry of Finance or Treasury may be relevant. Supporting decision makers’ assessments of whether an SHG is a good value requires assessing outcomes alongside costs (i.e. aggregating costs and outcomes). Where the studies assessed as part of this review aggregate costs and outcomes, they combine these into ICERs and, most frequently, compare the ICERs to a decision-making threshold based on GDP per capita. The decision-making threshold should reflect the opportunity cost of funding the intervention within the sector that would fund it, and where these are multiple, it should reflect each separately. While there is proof of concept that such a framework can be applied in analysis in an LMIC setting from Ramponi *et al.* (2022), it is nonetheless challenging to conduct such analyses both in terms of the additional analytical capacity and data requirements.

The current review builds on the existing literature that evaluates SHGs. Specifically, Gugerty *et al.* (2019) undertook an evidence review of the impacts of interventions delivered through SHGs on health and other relevant outcomes: finance, agriculture and empowerment outcomes in South Asia and sub-Saharan Africa. The current review expands upon this to consider all LMICs but takes a somewhat narrower focus looking specifically at studies that include both costs and effects (as opposed to effects only), given our aim being the identification and critical appraisal of the methods used to account for effects and/or costs falling across multiple sectors in evaluations of SHG interventions that seek to improve health, and potentially other outcomes, in LMICs.

## Conclusion

This paper considered whether the methods employed by studies evaluating SHG interventions that seek to improve, at least, health in LMICs can meaningfully inform decisions by ministries of health and other sectors, including donors, around whether to fund such interventions. Our findings suggest a mix in terms of the extent to which published studies are able to inform decision makers around the value of implementing SHG interventions in their given context. In particular, overall we found there to be potential value in improving the clarity with which the decision context is described or understood within most of the papers reviewed. Informing decisions around funding SHGs in a way that ensures that their value is accounted for requires collection and reporting of the costs and outcomes associated with providing an intervention, as well as understanding the opportunity cost of providing the intervention for each relevant sector. Therefore, this requires understanding the decision context, namely who the decision maker is (or who the decision makers are) and what decision(s) they are looking to inform. This informs the requirements for data collection around which costs and outcomes to include, and the relevant margin for opportunity costs, by sector, so these can be aggregated appropriately. Furthermore, organizing this evidence in a cross-sectoral framework enables different potential methods of aggregation, which may impact the ultimate recommendations and the incorporation of equity considerations.

## Supplementary Material

czad060_Supp

## Data Availability

All data generated or analysed during this study are included in this published article.

## Reflexivity statement

Given the nature of this study and the wider project within which it falls (NIHR RIGHT: Transforming the Treatment and Prevention of Leprosy and Buruli ulcers in LMICs), expertise was required around how SHGs as a clinical intervention to improve health outcomes are defined in order to ensure the review could fulfil its objective of informing methods for assessing SHG interventions with effects in and beyond health. Expertise was also required in best practice in methods for economic evaluation and best practice for conducting a scoping review. As such, the project team comprises individuals who work in leprosy and who understand, practically, what an SHG is: J.D., K.G., A.M., D.S., I.B.N. and R.J.L. J.O., N.K.G., R.F. and M.S. provided expertise in methods for conducting economic evaluation. M.H. provided expertise in methods for conducting a scoping review. A roundtable workshop that follows on from this study, organized by J.O. and N.K.G. and including presentations by D.S., M.S., J.O., N.K.G. and others, will gather input from J.O., N.K.G., J.D., K.G., A.M., D.S., I.B.N., R.J.L., M.S. and a wider range of experts in leprosy and economic evaluation to digest the results of this work to further inform best practice for evaluating SHG interventions.
