# Supplementary material for: Economic evaluation of self-help group interventions for health in LMICs: a scoping review
Source: Health Policy Plan. 2023 Jul 27;38(9):1033–49. doi: 10.1093/heapol/czad060 (PMC10566324; doi:10.1093/heapol/czad060)
Supplement: czad060_Supp [file czad060_supp.zip › suppl_data/Appendix.docx]

**Appendix**

**Search strategies**

- Search filters for LMIC countries developed by the Cochrane Effective Practice and Organisation of Care group were used in the MEDLINE, EMBASE, PsycINFO and EconLIt strategies.^1^
- A search strategy to limit retrieval to economic evaluations, developed by the Centre for Reviews and Dissemiation (CRD) was used in the search of MEDLINE, EMBASE and PsycINFO.^2^

**MEDLINE ALL**

(includes: Epub Ahead of Print, In-Process & Other Non-Indexed Citations, Ovid MEDLINE Daily and Ovid MEDLINE)

via Ovid <http://ovidsp.ovid.com/>

1946 to April 27, 2021

Searched on: 28^th^ April 2021

Records retrieved: 1993

1 (afghanistan or albania or algeria or american samoa or angola or "antigua and barbuda" or antigua or barbuda or argentina or armenia or aruba or azerbaijan or bahrain or bangladesh or barbados or republic of belarus or belarus or byelarus or belorussia or belize or british honduras or benin or dahomey or bhutan or bolivia or "bosnia and herzegovina" or bosnia or herzegovina or botswana or bechuanaland or brazil or brasil or bulgaria or burkina faso or burkina fasso or upper volta or burundi or urundi or cabo verde or cape verde or cambodia or kampuchea or khmer republic or cameroon or cameron or cameroun or central african republic or ubangi shari or chad or chile or china or colombia or comoros or comoro islands or iles comores or mayotte or democratic republic of the congo or democratic republic congo or congo or zaire or costa rica or "cote d’ivoire" or "cote d’ ivoire" or cote divoire or cote d ivoire or ivory coast or croatia or cuba or cyprus or czech republic or czechoslovakia or djibouti or french somaliland or dominica or dominican republic or ecuador or egypt or united arab republic or el salvador or equatorial guinea or spanish guinea or eritrea or estonia or eswatini or swaziland or ethiopia or fiji or gabon or gabonese republic or gambia or "georgia (republic)" or ghana or gold coast or gibraltar or greece or grenada or guam or guatemala or guinea or guinea bissau or guyana or british guiana or haiti or hispaniola or honduras or hungary or india or indonesia or timor or iran or iraq or isle of man or jamaica or jordan or kazakhstan or kazakh or kenya or "democratic people’s republic of korea" or republic of korea or north korea or south korea or korea or kosovo or kyrgyzstan or kirghizia or kirgizstan or kyrgyz republic or kirghiz or laos or lao pdr or "lao people's democratic republic" or latvia or lebanon or lebanese republic or lesotho or basutoland or liberia or libya or libyan arab jamahiriya or lithuania or macau or macao or "macedonia (republic)" or macedonia or madagascar or malagasy republic or malawi or nyasaland or malaysia or malay federation or malaya federation or maldives or indian ocean islands or indian ocean or mali or malta or micronesia or federated states of micronesia or kiribati or marshall islands or nauru or northern mariana islands or palau or tuvalu or mauritania or mauritius or mexico or moldova or moldovian or mongolia or montenegro or morocco or ifni or mozambique or portuguese east africa or myanmar or burma or namibia or nepal or netherlands antilles or nicaragua or niger or nigeria or oman or muscat or pakistan or panama or papua new guinea or new guinea or paraguay or peru or philippines or philipines or phillipines or phillippines or poland or "polish people's republic" or portugal or portuguese republic or puerto rico or romania or russia or russian federation or ussr or soviet union or union of soviet socialist republics or rwanda or ruanda or samoa or pacific islands or polynesia or samoan islands or navigator island or navigator islands or "sao tome and principe" or saudi arabia or senegal or serbia or seychelles or sierra leone or slovakia or slovak republic or slovenia or melanesia or solomon island or solomon islands or norfolk island or norfolk islands or somalia or south africa or south sudan or sri lanka or ceylon or "saint kitts and nevis" or "st. kitts and nevis" or saint lucia or "st. lucia" or "saint vincent and the grenadines" or saint vincent or "st. vincent" or grenadines or sudan or suriname or surinam or dutch guiana or netherlands guiana or syria or syrian arab republic or tajikistan or tadjikistan or tadzhikistan or tadzhik or taiwan or tanzania or tanganyika or thailand or siam or timor leste or east timor or togo or togolese republic or tonga or "trinidad and tobago" or trinidad or tobago or tunisia or turkey or "turkey (republic)" or turkmenistan or uganda or ukraine or uruguay or uzbekistan or vanuatu or new hebrides or venezuela or vietnam or viet nam or middle east or west bank or gaza or palestine or yemen or yugoslavia or zambia or zimbabwe or northern rhodesia or global south or africa south of the sahara or sub-saharan africa or subsaharan africa or africa, central or central africa or africa, northern or north africa or northern africa or magreb or maghrib or sahara or africa, southern or southern africa or africa, eastern or east africa or eastern africa or africa, western or west africa or western africa or west indies or indian ocean islands or caribbean or central america or latin america or "south and central america" or south america or asia, central or central asia or asia, northern or north asia or northern asia or asia, southeastern or southeastern asia or south eastern asia or southeast asia or south east asia or asia, western or western asia or europe, eastern or east europe or eastern europe or developing country or developing countries or developing nation? or developing population? or developing world or less developed countr* or less developed nation? or less developed population? or less developed world or lesser developed countr* or lesser developed nation? or lesser developed population? or lesser developed world or under developed countr* or under developed nation? or under developed population? or under developed world or underdeveloped countr* or underdeveloped nation? or underdeveloped population? or underdeveloped world or middle income countr* or middle income nation? or middle income population? or low income countr* or low income nation? or low income population? or lower income countr* or lower income nation? or lower income population? or underserved countr* or underserved nation? or underserved population? or underserved world or under served countr* or under served nation? or under served population? or under served world or deprived countr* or deprived nation? or deprived population? or deprived world or poor countr* or poor nation? or poor population? or poor world or poorer countr* or poorer nation? or poorer population? or poorer world or developing economy* or less developed economy* or lesser developed economy* or under developed economy* or underdeveloped economy* or middle income economy* or low income economy* or lower income economy* or low gdp or low gnp or low gross domestic or low gross national or lower gdp or lower gnp or lower gross domestic or lower gross national or lmic or lmics or third world or lami countr* or transitional countr* or emerging economies or emerging nation? or priority countr*).ti,ab,sh,kf. (2090014)

2 (afghan or afghans or afghani or albanian? or algerian? or american samoan? or angolan? or antiguan? or barbudan? or argentine? or argentinian? or argentinean? or armenian? or aruban? or azerbaijani? or bahraini? or bangladeshi? or bangalees or bajan? or belarusian? or byelorussian? or belizean? or beninese? or bhutanese or bolivian? or bosnian? or botswana or batswana or brazilian? or brasilian? or bulgarian? or burkinabe or burkinese or burundian? or cape verdean? or cabo verdean? or cambodian? or khmer or cameroonian? or central african? or chadian? or chilean? or chinese or colombian? or comorian? or congolese or costa rican? or ivorian? or croatian? or cuban? or cypriot? or czech? or djiboutian? or dominican? or ecuadorian? or egyptian? or salvadoran? or equatorial guinean? or equatoguinean? or eritrean? or estonian? or swazi? or swati? or ethiopian? or fijian or gabonese or gabonaise or gambian? or georgian? or ghanaian? or gibraltarian? or greek? or grenadian? or guamanian? or guatemalan? or guinean? or bissau guinean? or guyanese or haitian? or honduran? or hungarian? or indian? or indonesian? or iranian? or iraqian? or iraqi? or manx or jamaican? or jordanian? or kazakhstani? or kenyan? or kirabati or kirabatian? or north korean? or korean? or kosovar? or kosovan? or kyrgyz* or lao or laotian? or latvian? or lebanese or lesothan? or lesothonian? or mosotho or basotho or liberian? or libyan? or lithuanian? or macanese or macedonian? or malagasy or madagascan? or malawian? or malaysian? or maldivian? or malian? or maltese or marshallese? or mauritanian? or mauritian? or mexican? or micronesian? or moldovan? or moldovian? or mongolian? or mongol or montenegrin? or moroccan? or mozambican? or burmese or myanma or namibian? or nauruan? or nepali or nepalese or netherlands antillean? or nicaraguan? or nigerien? or nigerian? or northern mariana islander? or mariana? or omani? or pakistani? or palauan? or panamanian? or papua new guinean? or paraguayan? or peruvian? or philippine? or philipine? or phillipine? or phillippine? or filipino? or filipina? or polish or pole or poles or portuguese or puerto rican? or romanian? or russian? or soviet people or soviet population or rwandan? or rwandese or ruandan? or ruandese or samoan? or sao tomean? or santomean? or saudi arabian? or saudi? or senegalese or serbian? or montenegrin? or seychellois or seychelloise? or sierra leonean? or slovak? or slovene? or solomon islander? or somali? or south african? or south sudanese or sri lankan? or ceylonese or kittitian? or nevisian? or saint lucian? or vincentian? or sudanese or surinamese? or syrian? or taiwanese? or tajik? or tajikistani? or tanzanian? or tanganyikan? or thai or timorese? or togolese or tongan? or trinidadian? or tobagonian? or tunisian? or turk? or turkish or turkmen? or tuvaluan? or ugandan? or ukrainian? or uruguayan? or uzbek? or vanuatu* or venezuelan? or vietnamese or yemeni? or yemenite? or yemenese or yugoslav? or yugoslavian? or zambian? or zimbabwean?).ti,ab,sh,kf. (901704)

3 1 or 2 (2468790)

4 Self-Help Groups/ (9269)

5 Peer Group/ (21416)

6 ((selfhelp or self help) adj3 (group or groups)).ti,ab. (1972)

7 ((selfhelp or self help) adj3 support$).ti,ab. (357)

8 (SHG or SHGs).ti,ab. (3351)

9 (support$ adj2 (group or groups)).ti,ab. (13637)

10 (peer$ adj2 (group or groups)).ti,ab. (4319)

11 (peer$ adj2 support$).ti,ab. (6209)

12 ((women$ or woman$) adj2 (group or groups or club$ or cooperative$ or co-operative$ or committee$ or association$)).ti,ab. (25154)

13 ((women$ or woman$) adj2 (selfhelp or self help)).ti,ab. (80)

14 (mutual adj (aid or assist$)).ti,ab. (492)

15 ((advocacy or saving$) adj group$).ti,ab. (1594)

16 (ROSCA or ASCA or VSLA).ti,ab. (572)

17 ((rotating or accumulating or village) adj (saving$ or save$)).ti,ab. (19)

18 (village$ adj2 (group or groups or club$ or cooperative$ or co-operative$ or committee$ or association$)).ti,ab. (557)

19 or/4-18 (79294)

20 3 and 19 (12456)

21 Community Networks/ (7053)

22 Social Participation/ (2715)

23 Community Participation/ (17599)

24 (communit$ adj2 (group or groups)).ti,ab. (4934)

25 (communit$ adj2 program$).ti,ab. (9192)

26 (communit$ adj2 (intervention$ or rehabilitat$)).ti,ab. (9349)

27 (communi$ adj2 mobili$).ti,ab. (2123)

28 (communit$ adj2 participat$).ti,ab. (9214)

29 (communit$ adj2 club$).ti,ab. (201)

30 or/21-29 (55818)

31 3 and 30 (13798)

32 Financing, Organized/ (6885)

33 Financial Support/ (3828)

34 (microfinanc$ or micro financ$ or microcredit or micro credit or microsaving$ or micro saving$ or microloan$ or micro loan$ or microinsurance or micro insurance or microenterpris$ or micro enterpris$).ti,ab. (492)

35 income generat$.ti,ab. (916)

36 ((lending or loan$) adj3 (group or groups)).ti,ab. (78)

37 (livelihood$ adj (group or groups)).ti,ab. (10)

38 or/32-37 (11998)

39 3 and 38 (2921)

40 (return adj3 investment$).mp. (2433)

41 (SROI or ROI).mp. (11553)

42 40 or 41 (13602)

43 economics/ (27317)

44 exp "costs and cost analysis"/ (244352)

45 economics, dental/ (1917)

46 exp "economics, hospital"/ (25066)

47 economics, medical/ (9130)

48 economics, nursing/ (4002)

49 economics, pharmaceutical/ (2982)

50 (economic$ or cost$ or price or prices or pricing or pharmacoeconomic$).ti,ab. (893083)

51 (expenditure$ not energy).ti,ab. (31685)

52 value for money.ti,ab. (1811)

53 budget$.ti,ab. (30906)

54 or/43-53 (1048398)

55 ((energy or oxygen) adj cost).ti,ab. (4262)

56 (metabolic adj cost).ti,ab. (1486)

57 ((energy or oxygen) adj expenditure).ti,ab. (26145)

58 or/55-57 (30894)

59 54 not 58 (1041305)

60 42 or 59 (1052795)

61 letter.pt. (1132815)

62 editorial.pt. (565207)

63 historical article.pt. (363200)

64 or/61-63 (2041062)

65 60 not 64 (1014456)

66 20 and 65 (1722)

67 31 and 65 (2811)

68 39 and 65 (1221)

69 66 or 67 or 68 (5365)

70 exp animals/ not humans/ (4818096)

71 69 not 70 (5324)

72 limit 71 to yr="2014 -Current" (1993)

**Embase**

via Ovid <http://ovidsp.ovid.com/>

1974 to 2021 April 26

Searched on: 28^th^ April 2021

Records retrieved: 3381

1 (afghanistan or albania or algeria or american samoa or angola or "antigua and barbuda" or antigua or barbuda or argentina or armenia or armenian or aruba or azerbaijan or bahrain or bangladesh or barbados or republic of belarus or belarus or byelarus or belorussia or byelorussian or belize or british honduras or benin or dahomey or bhutan or bolivia or "bosnia and herzegovina" or bosnia or herzegovina or botswana or bechuanaland or brazil or brasil or bulgaria or burkina faso or burkina fasso or upper volta or burundi or urundi or cabo verde or cape verde or cambodia or kampuchea or khmer republic or cameroon or cameron or cameroun or central african republic or ubangi shari or chad or chile or china or colombia or comoros or comoro islands or iles comores or mayotte or democratic republic of the congo or democratic republic congo or congo or zaire or costa rica or "cote d’ivoire" or "cote d’ ivoire" or cote divoire or cote d ivoire or ivory coast or croatia or cuba or cyprus or czech republic or czechoslovakia or djibouti or french somaliland or dominica or dominican republic or ecuador or egypt or united arab republic or el salvador or equatorial guinea or spanish guinea or eritrea or estonia or eswatini or swaziland or ethiopia or fiji or gabon or gabonese republic or gambia or "georgia (republic)" or georgian or ghana or gold coast or gibraltar or greece or grenada or guam or guatemala or guinea or guinea bissau or guyana or british guiana or haiti or hispaniola or honduras or hungary or india or indonesia or timor or iran or iraq or isle of man or jamaica or jordan or kazakhstan or kazakh or kenya or "democratic people’s republic of korea" or republic of korea or north korea or south korea or korea or kosovo or kyrgyzstan or kirghizia or kirgizstan or kyrgyz republic or kirghiz or laos or lao pdr or "lao people's democratic republic" or latvia or lebanon or lebanese republic or lesotho or basutoland or liberia or libya or libyan arab jamahiriya or lithuania or macau or macao or republic of north macedonia or macedonia or madagascar or malagasy republic or malawi or nyasaland or malaysia or malay federation or malaya federation or maldives or indian ocean islands or indian ocean or mali or malta or micronesia or federated states of micronesia or kiribati or marshall islands or nauru or northern mariana islands or palau or tuvalu or mauritania or mauritius or mexico or moldova or moldovian or mongolia or montenegro or "montenegro (republic)" or morocco or ifni or mozambique or portuguese east africa or myanmar or burma or namibia or nepal or netherlands antilles or nicaragua or niger or nigeria or oman or muscat or pakistan or panama or papua new guinea or new guinea or paraguay or peru or philippines or philipines or phillipines or phillippines or poland or "polish people's republic" or portugal or portuguese republic or puerto rico or romania or russia or russian federation or ussr or soviet union or union of soviet socialist republics or rwanda or ruanda or samoa or pacific islands or polynesia or samoan islands or navigator island or navigator islands or "sao tome and principe" or saudi arabia or senegal or serbia or seychelles or sierra leone or slovakia or slovak republic or slovenia or melanesia or solomon island or solomon islands or norfolk island or norfolk islands or somalia or south africa or south sudan or sri lanka or ceylon or "saint kitts and nevis" or "st. kitts and nevis" or saint lucia or "st. lucia" or "saint vincent and the grenadines" or saint vincent or "st. vincent" or grenadines or sudan or suriname or surinam or dutch guiana or netherlands guiana or syria or syrian arab republic or taiwan or tajikistan or tadjikistan or tadzhikistan or tadzhik or tanzania or tanganyika or thailand or siam or timor leste or east timor or togo or togolese republic or tonga or "trinidad and tobago" or trinidad or tobago or tunisia or "turkey (republic)" or turkey or turkmenistan or turkmen or uganda or ukraine or uruguay or uzbekistan or uzbek or vanuatu or new hebrides or venezuela or vietnam or viet nam or middle east or west bank or gaza or palestine or yemen or yugoslavia or zambia or zimbabwe or northern rhodesia or global south or africa south of the sahara or "sub saharan africa" or subsaharan africa or africa, central or central africa or africa, northern or north africa or northern africa or magreb or maghrib or sahara or africa, southern or southern africa or africa, eastern or east africa or eastern africa or africa, western or west africa or western africa or west indies or indian ocean islands or caribbean region or caribbean islands or caribbean or central america or latin america or "south and central america" or south america or asia, central or central asia or asia, northern or north asia or northern asia or asia, southeastern or southeastern asia or south eastern asia or southeast asia or south east asia or asia, western or western asia or europe, eastern or east europe or eastern europe or developing country or developing countries or developing nation? or developing population? or developing world or less developed countr* or less developed nation? or less developed population? or less developed world or lesser developed countr* or lesser developed nation? or lesser developed population? or lesser developed world or under developed countr* or under developed nation? or under developed population? or under developed world or underdeveloped countr* or underdeveloped nation? or underdeveloped population? or underdeveloped world or middle income countr* or middle income nation? or middle income population? or low income countr* or low income nation? or low income population? or lower income countr* or lower income nation? or lower income population? or underserved countr* or underserved nation? or underserved population? or underserved world or under served countr* or under served nation? or under served population? or under served world or deprived countr* or deprived nation? or deprived population? or deprived world or poor countr* or poor nation? or poor population? or poor world or poorer countr* or poorer nation? or poorer population? or poorer world or developing econom* or less developed econom* or lesser developed econom* or under developed econom* or underdeveloped econom* or middle income econom* or low income econom* or lower income econom* or low gdp or low gnp or low gross domestic or low gross national or lower gdp or lower gnp or lower gross domestic or lower gross national or lmic or lmics or third world or lami countr* or transitional countr* or emerging economies or emerging nation? or priority countr*).ti,ab,sh,kw. (2399705)

2 (afghan or afghans or afghani or albanian? or algerian? or american samoan? or angolan? or antiguan? or barbudan? or argentine? or argentinian? or argentinean? or armenian? or aruban? or azerbaijani? or bahraini? or bangladeshi? or bangalees or bajan? or belarusian? or byelorussian? or belizean? or beninese? or bhutanese or bolivian? or bosnian? or botswana or batswana or brazilian? or brasilian? or bulgarian? or burkinabe or burkinese or burundian? or cape verdean? or cabo verdean? or cambodian? or khmer or cameroonian? or central african? or chadian? or chilean? or chinese or colombian? or comorian? or congolese or costa rican? or ivorian? or croatian? or cuban? or cypriot? or czech? or djiboutian? or dominican? or ecuadorian? or egyptian? or salvadoran? or equatorial guinean? or equatoguinean? or eritrean? or estonian? or swazi? or swati? or ethiopian? or fijian or gabonese or gabonaise or gambian? or georgian? or ghanaian? or gibraltarian? or greek? or grenadian? or guamanian? or guatemalan? or guinean? or bissau guinean? or guyanese or haitian? or honduran? or hungarian? or indian? or indonesian? or iranian? or iraqian? or iraqi? or manx or jamaican? or jordanian? or kazakhstani? or kenyan? or kirabati or kirabatian? or north korean? or korean? or kosovar? or kosovan? or kyrgyz* or lao or laotian? or latvian? or lebanese or lesothan? or lesothonian? or mosotho or basotho or liberian? or libyan? or lithuanian? or macanese or macedonian? or malagasy or madagascan? or malawian? or malaysian? or maldivian? or malian? or maltese or marshallese? or mauritanian? or mauritian? or mexican? or micronesian? or moldovan? or mongolian? or mongol or montenegrin? or moroccan? or mozambican? or burmese or myanma or namibian? or nauruan? or nepali or nepalese or netherlands antillean? or nicaraguan? or nigerien? or nigerian? or northern mariana islander? or mariana? or omani? or pakistani? or palauan? or panamanian? or papua new guinean? or paraguayan? or peruvian? or philippine? or philipine? or phillipine? or phillippine? or filipino? or filipina? or polish or pole or poles or portuguese or puerto rican? or romanian? or russian? or soviet people or soviet population or rwandan? or rwandese or ruandan? or ruandese or samoan? or sao tomean? or santomean? or saudi arabian? or saudi? or senegalese or serbian? or montenegrin? or seychellois or seychelloise? or sierra leonean? or slovak? or slovene? or solomon islander? or somali? or south african? or south sudanese or sri lankan? or ceylonese or kittitian? or nevisian? or saint lucian? or vincentian? or sudanese or surinamese? or syrian? or taiwanese or tajik? or tajikistani? or tanzanian? or tanganyikan? or thai or timorese? or togolese or tongan? or trinidadian? or tobagonian? or tunisian? or turk? or turkish or turkmen? or tuvaluan? or ugandan? or ukrainian? or uruguayan? or uzbek? or vanuatu* or venezuelan? or vietnamese or yemeni? or yemenite? or yemenese or yugoslav? or yugoslavian? or zambian? or zimbabwean?).ti,ab,sh,kw. (1231924)

3 1 or 2 (3030193)

4 self help/ (13970)

5 peer group/ (25337)

6 support group/ (12764)

7 ((selfhelp or self help) adj3 (group or groups)).ti,ab. (2694)

8 ((selfhelp or self help) adj3 support$).ti,ab. (489)

9 (SHG or SHGs).ti,ab. (3424)

10 (support$ adj2 (group or groups)).ti,ab. (20186)

11 (peer$ adj2 (group or groups)).ti,ab. (5871)

12 (peer$ adj2 support$).ti,ab. (8756)

13 ((women$ or woman$) adj2 (group or groups or club$ or cooperative$ or co-operative$ or committee$ or association$)).ti,ab. (36028)

14 ((women$ or woman$) adj2 (selfhelp or self help)).ti,ab. (94)

15 (mutual adj (aid or assist$)).ti,ab. (602)

16 ((advocacy or saving$) adj group$).ti,ab. (2474)

17 (ROSCA or ASCA or VSLA).ti,ab. (1157)

18 ((rotating or accumulating or village) adj (saving$ or save$)).ti,ab. (27)

19 (village$ adj2 (group or groups or club$ or cooperative$ or co-operative$ or committee$ or association$)).ti,ab. (598)

20 or/4-19 (112323)

21 3 and 20 (16677)

22 *community care/ (21188)

23 community based rehabilitation/ (823)

24 community program/ (2949)

25 social participation/ (7162)

26 community participation/ (2954)

27 (communit$ adj2 (group or groups)).ti,ab. (6502)

28 (communit$ adj2 program$).ti,ab. (12043)

29 (communit$ adj2 (intervention$ or rehabilitat$)).ti,ab. (12533)

30 (communi$ adj2 mobili$).ti,ab. (2676)

31 (communit$ adj2 participat$).ti,ab. (11041)

32 (communit$ adj2 club$).ti,ab. (254)

33 or/22-32 (70560)

34 3 and 33 (18190)

35 *financial management/ (45116)

36 (microfinanc$ or micro financ$ or microcredit or micro credit or microsaving$ or micro saving$ or microloan$ or micro loan$ or microinsurance or micro insurance or microenterpris$ or micro enterpris$).ti,ab. (551)

37 income generat$.ti,ab. (905)

38 ((lending or loan$) adj3 (group or groups)).ti,ab. (87)

39 (livelihood$ adj (group or groups)).ti,ab. (9)

40 35 or 36 or 37 or 38 or 39 (46507)

41 3 and 40 (4500)

42 (return adj3 investment$).mp. (3246)

43 (SROI or ROI).mp. (21329)

44 42 or 43 (24007)

45 Health Economics/ (33920)

46 exp Economic Evaluation/ (322612)

47 exp Health Care Cost/ (307579)

48 pharmacoeconomics/ (8641)

49 45 or 46 or 47 or 48 (567746)

50 (econom$ or cost or costs or costly or costing or price or prices or pricing or pharmacoeconomic$).ti,ab. (1161726)

51 (expenditure$ not energy).ti,ab. (43666)

52 (value adj2 money).ti,ab. (2600)

53 budget$.ti,ab. (41282)

54 50 or 51 or 52 or 53 (1200701)

55 44 or 49 or 54 (1456329)

56 letter.pt. (1200828)

57 editorial.pt. (701383)

58 note.pt. (864777)

59 56 or 57 or 58 (2766988)

60 55 not 59 (1345780)

61 (metabolic adj cost).ti,ab. (1618)

62 ((energy or oxygen) adj cost).ti,ab. (4573)

63 ((energy or oxygen) adj expenditure).ti,ab. (33678)

64 61 or 62 or 63 (38725)

65 60 not 64 (1337904)

66 21 and 65 (2049)

67 34 and 65 (3477)

68 41 and 65 (1742)

69 66 or 67 or 68 (6913)

70 (animal/ or animal experiment/ or animal model/ or animal tissue/ or nonhuman/) not exp human/ (6297408)

71 69 not 70 (6838)

72 limit 71 to yr="2014 -Current" (3381)

73 (conference abstract or "conference review").pt. (4093387)

74 72 and 73 (729)

75 72 not 73 (2652)

**PsycINFO**

via Ovid <http://ovidsp.ovid.com/>

1806 to April Week 3 2021

Searched on: 28^th^ April 2021

Records retrieved: 117

1 (afghanistan or albania or algeria or american samoa or angola or "antigua and barbuda" or antigua or barbuda or argentina or armenia or aruba or azerbaijan or bahrain or bangladesh or barbados or republic of belarus or belarus or byelarus or belorussia or belize or british honduras or benin or dahomey or bhutan or bolivia or "bosnia and herzegovina" or bosnia or herzegovina or botswana or bechuanaland or brazil or brasil or bulgaria or burkina faso or burkina fasso or upper volta or burundi or urundi or cabo verde or cape verde or cambodia or kampuchea or khmer republic or cameroon or cameron or cameroun or central african republic or ubangi shari or chad or chile or china or colombia or comoros or comoro islands or iles comores or mayotte or democratic republic of the congo or democratic republic congo or congo or zaire or costa rica or "cote d’ivoire" or "cote d’ ivoire" or cote divoire or cote d ivoire or ivory coast or croatia or cuba or cyprus or czech republic or czechoslovakia or djibouti or french somaliland or dominica or dominican republic or ecuador or egypt or united arab republic or el salvador or equatorial guinea or spanish guinea or eritrea or estonia or eswatini or swaziland or ethiopia or fiji or gabon or gabonese republic or gambia or "georgia (republic)" or ghana or gold coast or gibraltar or greece or grenada or guam or guatemala or guinea or guinea bissau or guyana or british guiana or haiti or hispaniola or honduras or hungary or india or indonesia or timor or iran or iraq or isle of man or jamaica or jordan or kazakhstan or kazakh or kenya or "democratic people’s republic of korea" or republic of korea or north korea or south korea or korea or kosovo or kyrgyzstan or kirghizia or kirgizstan or kyrgyz republic or kirghiz or laos or lao pdr or "lao people's democratic republic" or latvia or lebanon or lebanese republic or lesotho or basutoland or liberia or libya or libyan arab jamahiriya or lithuania or macau or macao or "macedonia (republic)" or macedonia or madagascar or malagasy republic or malawi or nyasaland or malaysia or malay federation or malaya federation or maldives or indian ocean islands or indian ocean or mali or malta or micronesia or federated states of micronesia or kiribati or marshall islands or nauru or northern mariana islands or palau or tuvalu or mauritania or mauritius or mexico or moldova or moldovian or mongolia or montenegro or morocco or ifni or mozambique or portuguese east africa or myanmar or burma or namibia or nepal or netherlands antilles or nicaragua or niger or nigeria or oman or muscat or pakistan or panama or papua new guinea or new guinea or paraguay or peru or philippines or philipines or phillipines or phillippines or poland or "polish people's republic" or portugal or portuguese republic or puerto rico or romania or russia or russian federation or ussr or soviet union or union of soviet socialist republics or rwanda or ruanda or samoa or pacific islands or polynesia or samoan islands or navigator island or navigator islands or "sao tome and principe" or saudi arabia or senegal or serbia or seychelles or sierra leone or slovakia or slovak republic or slovenia or melanesia or solomon island or solomon islands or norfolk island or norfolk islands or somalia or south africa or south sudan or sri lanka or ceylon or "saint kitts and nevis" or "st. kitts and nevis" or saint lucia or "st. lucia" or "saint vincent and the grenadines" or saint vincent or "st. vincent" or grenadines or sudan or suriname or surinam or dutch guiana or netherlands guiana or syria or syrian arab republic or tajikistan or tadjikistan or tadzhikistan or tadzhik or taiwan or tanzania or tanganyika or thailand or siam or timor leste or east timor or togo or togolese republic or tonga or "trinidad and tobago" or trinidad or tobago or tunisia or turkey or "turkey (republic)" or turkmenistan or uganda or ukraine or uruguay or uzbekistan or vanuatu or new hebrides or venezuela or vietnam or viet nam or middle east or west bank or gaza or palestine or yemen or yugoslavia or zambia or zimbabwe or northern rhodesia or global south or africa south of the sahara or sub-saharan africa or subsaharan africa or africa, central or central africa or africa, northern or north africa or northern africa or magreb or maghrib or sahara or africa, southern or southern africa or africa, eastern or east africa or eastern africa or africa, western or west africa or western africa or west indies or indian ocean islands or caribbean or central america or latin america or "south and central america" or south america or asia, central or central asia or asia, northern or north asia or northern asia or asia, southeastern or southeastern asia or south eastern asia or southeast asia or south east asia or asia, western or western asia or europe, eastern or east europe or eastern europe or developing country or developing countries or developing nation? or developing population? or developing world or less developed countr* or less developed nation? or less developed population? or less developed world or lesser developed countr* or lesser developed nation? or lesser developed population? or lesser developed world or under developed countr* or under developed nation? or under developed population? or under developed world or underdeveloped countr* or underdeveloped nation? or underdeveloped population? or underdeveloped world or middle income countr* or middle income nation? or middle income population? or low income countr* or low income nation? or low income population? or lower income countr* or lower income nation? or lower income population? or underserved countr* or underserved nation? or underserved population? or underserved world or under served countr* or under served nation? or under served population? or under served world or deprived countr* or deprived nation? or deprived population? or deprived world or poor countr* or poor nation? or poor population? or poor world or poorer countr* or poorer nation? or poorer population? or poorer world or developing economy* or less developed economy* or lesser developed economy* or under developed economy* or underdeveloped economy* or middle income economy* or low income economy* or lower income economy* or low gdp or low gnp or low gross domestic or low gross national or lower gdp or lower gnp or lower gross domestic or lower gross national or lmic or lmics or third world or lami countr* or transitional countr* or emerging economies or emerging nation? or priority countr*).ti,ab,sh,lo,id. (403558)

2 (afghan or afghans or afghani or albanian? or algerian? or american samoan? or angolan? or antiguan? or barbudan? or argentine? or argentinian? or argentinean? or armenian? or aruban? or azerbaijani? or bahraini? or bangladeshi? or bangalees or bajan? or belarusian? or byelorussian? or belizean? or beninese? or bhutanese or bolivian? or bosnian? or botswana or batswana or brazilian? or brasilian? or bulgarian? or burkinabe or burkinese or burundian? or cape verdean? or cabo verdean? or cambodian? or khmer or cameroonian? or central african? or chadian? or chilean? or chinese or colombian? or comorian? or congolese or costa rican? or ivorian? or croatian? or cuban? or cypriot? or czech? or djiboutian? or dominican? or ecuadorian? or egyptian? or salvadoran? or equatorial guinean? or equatoguinean? or eritrean? or estonian? or swazi? or swati? or ethiopian? or fijian or gabonese or gabonaise or gambian? or georgian? or ghanaian? or gibraltarian? or greek? or grenadian? or guamanian? or guatemalan? or guinean? or bissau guinean? or guyanese or haitian? or honduran? or hungarian? or indian? or indonesian? or iranian? or iraqian? or iraqi? or manx or jamaican? or jordanian? or kazakhstani? or kenyan? or kirabati or kirabatian? or north korean? or korean? or kosovar? or kosovan? or kyrgyz* or lao or laotian? or latvian? or lebanese or lesothan? or lesothonian? or mosotho or basotho or liberian? or libyan? or lithuanian? or macanese or macedonian? or malagasy or madagascan? or malawian? or malaysian? or maldivian? or malian? or maltese or marshallese? or mauritanian? or mauritian? or mexican? or micronesian? or moldovan? or moldovian? or mongolian? or mongol or montenegrin? or moroccan? or mozambican? or burmese or myanma or namibian? or nauruan? or nepali or nepalese or netherlands antillean? or nicaraguan? or nigerien? or nigerian? or northern mariana islander? or mariana? or omani? or pakistani? or palauan? or panamanian? or papua new guinean? or paraguayan? or peruvian? or philippine? or philipine? or phillipine? or phillippine? or filipino? or filipina? or polish or pole or poles or portuguese or puerto rican? or romanian? or russian? or soviet people or soviet population or rwandan? or rwandese or ruandan? or ruandese or samoan? or sao tomean? or santomean? or saudi arabian? or saudi? or senegalese or serbian? or montenegrin? or seychellois or seychelloise? or sierra leonean? or slovak? or slovene? or solomon islander? or somali? or south african? or south sudanese or sri lankan? or ceylonese or kittitian? or nevisian? or saint lucian? or vincentian? or sudanese or surinamese? or syrian? or taiwanese? or tajik? or tajikistani? or tanzanian? or tanganyikan? or thai or timorese? or togolese or tongan? or trinidadian? or tobagonian? or tunisian? or turk? or turkish or turkmen? or tuvaluan? or ugandan? or ukrainian? or uruguayan? or uzbek? or vanuatu* or venezuelan? or vietnamese or yemeni? or yemenite? or yemenese or yugoslav? or yugoslavian? or zambian? or zimbabwean?).ti,ab,sh,lo,id. (245419)

3 1 or 2 (492881)

4 self-help techniques/ (4283)

5 support groups/ (4340)

6 social support/ (38000)

7 peer relations/ or peers/ (29237)

8 ((selfhelp or self help) adj3 (group or groups)).ti,ab. (2220)

9 ((selfhelp or self help) adj3 support$).ti,ab. (448)

10 (SHG or SHGs).ti,ab. (92)

11 (support$ adj2 (group or groups)).ti,ab. (12842)

12 (peer$ adj2 (group or groups)).ti,ab. (7922)

13 (peer$ adj2 support$).ti,ab. (6321)

14 ((women$ or woman$) adj2 (group or groups or club$ or cooperative$ or co-operative$ or committee$ or association$)).ti,ab. (7280)

15 ((women$ or woman$) adj2 (selfhelp or self help)).ti,ab. (53)

16 (mutual adj (aid or assist$)).ti,ab. (771)

17 ((advocacy or saving$) adj group$).ti,ab. (984)

18 (ROSCA or ASCA or VSLA).ti,ab. (403)

19 ((rotating or accumulating or village) adj (saving$ or save$)).ti,ab. (15)

20 (village$ adj2 (group or groups or club$ or cooperative$ or co-operative$ or committee$ or association$)).ti,ab. (96)

21 or/4-20 (99060)

22 3 and 21 (12918)

23 social networks/ (12511)

24 community involvement/ (5277)

25 (communit$ adj2 (group or groups)).ti,ab. (4188)

26 (communit$ adj2 program$).ti,ab. (6689)

27 (communit$ adj2 (intervention$ or rehabilitat$)).ti,ab. (5472)

28 (communi$ adj2 mobili$).ti,ab. (1059)

29 (communit$ adj2 participat$).ti,ab. (5909)

30 (communit$ adj2 club$).ti,ab. (160)

31 or/23-30 (38418)

32 3 and 31 (6661)

33 finance/ (4702)

34 (microfinanc$ or micro financ$ or microcredit or micro credit or microsaving$ or micro saving$ or microloan$ or micro loan$ or microinsurance or micro insurance or microenterpris$ or micro enterpris$).ti,ab,id. (592)

35 income generat$.ti,ab. (374)

36 ((lending or loan$) adj3 (group or groups)).ti,ab. (72)

37 (livelihood$ adj (group or groups)).ti,ab. (0)

38 or/33-37 (5539)

39 3 and 38 (1308)

40 (return adj3 investment$).mp. (1211)

41 (SROI or ROI).mp. (2159)

42 40 or 41 (3122)

43 "costs and cost analysis"/ (17420)

44 "Cost Containment"/ (647)

45 (economic adj2 evaluation$).ti,ab. (1855)

46 (economic adj2 analy$).ti,ab. (1606)

47 (economic adj2 (study or studies)).ti,ab. (853)

48 (cost adj2 evaluation$).ti,ab. (358)

49 (cost adj2 analy$).ti,ab. (3927)

50 (cost adj2 (study or studies)).ti,ab. (923)

51 (cost adj2 effective$).ti,ab. (16177)

52 (cost adj2 benefit$).ti,ab. (3653)

53 (cost adj2 utili$).ti,ab. (1363)

54 (cost adj2 minimi$).ti,ab. (385)

55 (cost adj2 consequence$).ti,ab. (124)

56 (cost adj2 comparison$).ti,ab. (193)

57 (cost adj2 identificat$).ti,ab. (26)

58 (pharmacoeconomic$ or pharmaco-economic$).ti,ab. (325)

59 or/43-58 (36821)

60 (task adj2 cost$).ti,ab,id. (692)

61 (switch$ adj2 cost$).ti,ab,id. (1423)

62 (metabolic adj cost).ti,ab,id. (104)

63 ((energy or oxygen) adj cost).ti,ab,id. (297)

64 ((energy or oxygen) adj expenditure).ti,ab,id. (2828)

65 or/60-64 (5045)

66 editorial.dt. (44223)

67 letter.dt. (23567)

68 66 or 67 (67790)

69 65 or 68 (72811)

70 42 or 59 (39686)

71 70 not 69 (38812)

72 22 and 71 (85)

73 32 and 71 (85)

74 39 and 71 (62)

75 72 or 73 or 74 (216)

76 limit 75 to yr="2014 -Current" (117)

**EconLit**

via Ovid <http://ovidsp.ovid.com/>

1886 to April 22, 2021

Searched on: 28^th^ April 2021

Records retrieved: 467

1 (afghanistan or albania or algeria or american samoa or angola or "antigua and barbuda" or antigua or barbuda or argentina or armenia or aruba or azerbaijan or bahrain or bangladesh or barbados or republic of belarus or belarus or byelarus or belorussia or belize or british honduras or benin or dahomey or bhutan or bolivia or "bosnia and herzegovina" or bosnia or herzegovina or botswana or bechuanaland or brazil or brasil or bulgaria or burkina faso or burkina fasso or upper volta or burundi or urundi or cabo verde or cape verde or cambodia or kampuchea or khmer republic or cameroon or cameron or cameroun or central african republic or ubangi shari or chad or chile or china or colombia or comoros or comoro islands or iles comores or mayotte or democratic republic of the congo or democratic republic congo or congo or zaire or costa rica or "cote d’ivoire" or "cote d’ ivoire" or cote divoire or cote d ivoire or ivory coast or croatia or cuba or cyprus or czech republic or czechoslovakia or djibouti or french somaliland or dominica or dominican republic or ecuador or egypt or united arab republic or el salvador or equatorial guinea or spanish guinea or eritrea or estonia or eswatini or swaziland or ethiopia or fiji or gabon or gabonese republic or gambia or "georgia (republic)" or ghana or gold coast or gibraltar or greece or grenada or guam or guatemala or guinea or guinea bissau or guyana or british guiana or haiti or hispaniola or honduras or hungary or india or indonesia or timor or iran or iraq or isle of man or jamaica or jordan or kazakhstan or kazakh or kenya or "democratic people’s republic of korea" or republic of korea or north korea or south korea or korea or kosovo or kyrgyzstan or kirghizia or kirgizstan or kyrgyz republic or kirghiz or laos or lao pdr or "lao people's democratic republic" or latvia or lebanon or lebanese republic or lesotho or basutoland or liberia or libya or libyan arab jamahiriya or lithuania or macau or macao or "macedonia (republic)" or macedonia or madagascar or malagasy republic or malawi or nyasaland or malaysia or malay federation or malaya federation or maldives or indian ocean islands or indian ocean or mali or malta or micronesia or federated states of micronesia or kiribati or marshall islands or nauru or northern mariana islands or palau or tuvalu or mauritania or mauritius or mexico or moldova or moldovian or mongolia or montenegro or morocco or ifni or mozambique or portuguese east africa or myanmar or burma or namibia or nepal or netherlands antilles or nicaragua or niger or nigeria or oman or muscat or pakistan or panama or papua new guinea or new guinea or paraguay or peru or philippines or philipines or phillipines or phillippines or poland or "polish people's republic" or portugal or portuguese republic or puerto rico or romania or russia or russian federation or ussr or soviet union or union of soviet socialist republics or rwanda or ruanda or samoa or pacific islands or polynesia or samoan islands or navigator island or navigator islands or "sao tome and principe" or saudi arabia or senegal or serbia or seychelles or sierra leone or slovakia or slovak republic or slovenia or melanesia or solomon island or solomon islands or norfolk island or norfolk islands or somalia or south africa or south sudan or sri lanka or ceylon or "saint kitts and nevis" or "st. kitts and nevis" or saint lucia or "st. lucia" or "saint vincent and the grenadines" or saint vincent or "st. vincent" or grenadines or sudan or suriname or surinam or dutch guiana or netherlands guiana or syria or syrian arab republic or tajikistan or tadjikistan or tadzhikistan or tadzhik or taiwan or tanzania or tanganyika or thailand or siam or timor leste or east timor or togo or togolese republic or tonga or "trinidad and tobago" or trinidad or tobago or tunisia or turkey or "turkey (republic)" or turkmenistan or uganda or ukraine or uruguay or uzbekistan or vanuatu or new hebrides or venezuela or vietnam or viet nam or middle east or west bank or gaza or palestine or yemen or yugoslavia or zambia or zimbabwe or northern rhodesia or global south or africa south of the sahara or sub-saharan africa or subsaharan africa or africa, central or central africa or africa, northern or north africa or northern africa or magreb or maghrib or sahara or africa, southern or southern africa or africa, eastern or east africa or eastern africa or africa, western or west africa or western africa or west indies or indian ocean islands or caribbean or central america or latin america or "south and central america" or south america or asia, central or central asia or asia, northern or north asia or northern asia or asia, southeastern or southeastern asia or south eastern asia or southeast asia or south east asia or asia, western or western asia or europe, eastern or east europe or eastern europe or developing country or developing countries or developing nation? or developing population? or developing world or less developed countr* or less developed nation? or less developed population? or less developed world or lesser developed countr* or lesser developed nation? or lesser developed population? or lesser developed world or under developed countr* or under developed nation? or under developed population? or under developed world or underdeveloped countr* or underdeveloped nation? or underdeveloped population? or underdeveloped world or middle income countr* or middle income nation? or middle income population? or low income countr* or low income nation? or low income population? or lower income countr* or lower income nation? or lower income population? or underserved countr* or underserved nation? or underserved population? or underserved world or under served countr* or under served nation? or under served population? or under served world or deprived countr* or deprived nation? or deprived population? or deprived world or poor countr* or poor nation? or poor population? or poor world or poorer countr* or poorer nation? or poorer population? or poorer world or developing economy* or less developed economy* or lesser developed economy* or under developed economy* or underdeveloped economy* or middle income economy* or low income economy* or lower income economy* or low gdp or low gnp or low gross domestic or low gross national or lower gdp or lower gnp or lower gross domestic or lower gross national or lmic or lmics or third world or lami countr* or transitional countr* or emerging economies or emerging nation? or priority countr*).ti,ab,sh,ct,gr,kw. (391950)

2 (afghan or afghans or afghani or albanian? or algerian? or american samoan? or angolan? or antiguan? or barbudan? or argentine? or argentinian? or argentinean? or armenian? or aruban? or azerbaijani? or bahraini? or bangladeshi? or bangalees or bajan? or belarusian? or byelorussian? or belizean? or beninese? or bhutanese or bolivian? or bosnian? or botswana or batswana or brazilian? or brasilian? or bulgarian? or burkinabe or burkinese or burundian? or cape verdean? or cabo verdean? or cambodian? or khmer or cameroonian? or central african? or chadian? or chilean? or chinese or colombian? or comorian? or congolese or costa rican? or ivorian? or croatian? or cuban? or cypriot? or czech? or djiboutian? or dominican? or ecuadorian? or egyptian? or salvadoran? or equatorial guinean? or equatoguinean? or eritrean? or estonian? or swazi? or swati? or ethiopian? or fijian or gabonese or gabonaise or gambian? or georgian? or ghanaian? or gibraltarian? or greek? or grenadian? or guamanian? or guatemalan? or guinean? or bissau guinean? or guyanese or haitian? or honduran? or hungarian? or indian? or indonesian? or iranian? or iraqian? or iraqi? or manx or jamaican? or jordanian? or kazakhstani? or kenyan? or kirabati or kirabatian? or north korean? or korean? or kosovar? or kosovan? or kyrgyz* or lao or laotian? or latvian? or lebanese or lesothan? or lesothonian? or mosotho or basotho or liberian? or libyan? or lithuanian? or macanese or macedonian? or malagasy or madagascan? or malawian? or malaysian? or maldivian? or malian? or maltese or marshallese? or mauritanian? or mauritian? or mexican? or micronesian? or moldovan? or moldovian? or mongolian? or mongol or montenegrin? or moroccan? or mozambican? or burmese or myanma or namibian? or nauruan? or nepali or nepalese or netherlands antillean? or nicaraguan? or nigerien? or nigerian? or northern mariana islander? or mariana? or omani? or pakistani? or palauan? or panamanian? or papua new guinean? or paraguayan? or peruvian? or philippine? or philipine? or phillipine? or phillippine? or filipino? or filipina? or polish or pole or poles or portuguese or puerto rican? or romanian? or russian? or soviet people or soviet population or rwandan? or rwandese or ruandan? or ruandese or samoan? or sao tomean? or santomean? or saudi arabian? or saudi? or senegalese or serbian? or montenegrin? or seychellois or seychelloise? or sierra leonean? or slovak? or slovene? or solomon islander? or somali? or south african? or south sudanese or sri lankan? or ceylonese or kittitian? or nevisian? or saint lucian? or vincentian? or sudanese or surinamese? or syrian? or taiwanese? or tajik? or tajikistani? or tanzanian? or tanganyikan? or thai or timorese? or togolese or tongan? or trinidadian? or tobagonian? or tunisian? or turk? or turkish or turkmen? or tuvaluan? or ugandan? or ukrainian? or uruguayan? or uzbek? or vanuatu* or venezuelan? or vietnamese or yemeni? or yemenite? or yemenese or yugoslav? or yugoslavian? or zambian? or zimbabwean?).ti,ab,sh,ct,gr,kw. (130344)

3 1 or 2 (408063)

4 ((selfhelp or self help) adj3 (group or groups)).mp. (264)

5 ((selfhelp or self help) adj3 support$).mp. (8)

6 (SHG or SHGs).mp. (198)

7 (support$ adj2 (group or groups)).mp. (299)

8 (peer$ adj2 (group or groups)).mp. (654)

9 (peer$ adj2 support$).mp. (54)

10 ((women$ or woman$) adj2 (group or groups or club$ or cooperative$ or co-operative$ or committee$ or association$)).mp. (551)

11 ((women$ or woman$) adj2 (selfhelp or self help)).mp. (40)

12 (mutual adj (aid or assist$)).mp. (132)

13 ((advocacy or saving$) adj group$).mp. (133)

14 (ROSCA or ASCA or VSLA).mp. (55)

15 ((rotating or accumulating or village) adj (saving$ or save$)).mp. (96)

16 (village$ adj2 (group or groups or club$ or cooperative$ or co-operative$ or committee$ or association$)).mp. (123)

17 or/4-16 (2317)

18 3 and 17 (1070)

19 (communit$ adj2 (group or groups)).mp. (357)

20 (communit$ adj2 program$).mp. (396)

21 (communit$ adj2 (intervention$ or rehabilitat$)).mp. (135)

22 (communi$ adj2 mobili$).mp. (141)

23 (communit$ adj2 participat$).mp. (698)

24 (communit$ adj2 club$).mp. (9)

25 or/19-24 (1641)

26 3 and 25 (798)

27 (microfinanc$ or micro financ$ or microcredit or micro credit or microsaving$ or micro saving$ or microloan$ or micro loan$ or microinsurance or micro insurance or microenterpris$ or micro enterpris$).mp. (57624)

28 income generat$.mp. (882)

29 ((lending or loan$) adj3 (group or groups)).mp. (316)

30 (livelihood$ adj (group or groups)).mp. (10)

31 or/27-30 (58486)

32 3 and 31 (17963)

33 18 or 26 or 32 (19483)

34 I1$.cc. or health.mp. (113575)

35 33 and 34 (923)

36 limit 35 to yr="2014 -Current" (467)

**Global Index Medicus**

<https://www.globalindexmedicus.net/>

Searched on: 29^th^ April 2021

Records retrieved: 272 hits in total (249 after deduplication)

All available indexes searched:

- [African Index Medicus – AIM](http://indexmedicus.afro.who.int/)(AFRO/WHO)
- [Scientific and Technical Literature of Latin America and the Caribbean – LILACS](http://lilacs.bvsalud.org/en/)(AMRO-PAHO/WHO[,](http://lilacs.bvsalud.org/en/)by its specialized center BIREME)
- [Index Medicus for Eastern Mediterranean Region – IMEMR](http://www.emro.who.int/e-library/imemr/index.html) (EMRO/WHO)
- [Index Medicus for South-East Asia Region – IMSEAR](http://imsear.searo.who.int/)(SEARO/WHO)
- [Western Pacific Region Index Medicus – WPRIM](http://www.wprim.org/)(WPRO/WHO)

Advanced search:

1. (tw:("Self-Help Groups" OR "selfhelp group" OR "selfhelp groups" OR "self help group" OR "self help groups" OR "self-help group" OR "selfhelp support" OR "self help support" OR "self-help support" OR shg OR shgs OR "support group" OR "support groups" OR "peer group" OR "peer groups" OR "peer support" OR "mutual aid" OR "mutual assistance")) AND (tw:("Costs and Cost Analysis" OR "Cost-Benefit Analysis" OR economic* OR cost* OR price OR prices OR pricing OR pharmacoeconomic* OR expenditure* OR "value for money" OR budget* OR "return on investment" OR roi OR sroi )) 2014-2021 - 59 hits

2. (ti:("women's group" OR "womens group" OR "women's groups" OR "womens groups")) AND (tw:(economic* OR cost* OR price OR prices OR pricing OR pharmacoeconomic* OR expenditure* OR "value for money" OR budget* OR "return on investment" OR roi OR sroi)) 2014-2021 - 0 hits

3. (ab:("women's group" OR "womens group" OR "women's groups" OR "womens groups")) AND (tw:(economic* OR cost* OR price OR prices OR pricing OR pharmacoeconomic* OR expenditure* OR "value for money" OR budget* OR "return on investment" OR roi OR sroi)) 2014-2021 – 5 hits

4. (mh:("Community Networks" OR "Social Participation" OR "Community Participation" )) AND (tw:(economic* OR cost* OR price OR prices OR pricing OR pharmacoeconomic* OR expenditure* OR "value for money" OR budget* OR "return on investment" OR roi OR sroi)) 2014-2021 – 94 hits

5. (ti:("community group" OR "community groups" OR "community program" OR "community programs" OR "community programme" OR "community programmes" OR "community intervention" OR "community interventions" OR "community rehabilitation" OR "community mobilisation" OR "community mobilization" OR "community club" OR "community clubs" )) AND (tw:(economic* OR cost* OR price OR prices OR pricing OR pharmacoeconomic* OR expenditure* OR "value for money" OR budget* OR "return on investment" OR roi OR sroi)) 2014-2021 – 6 hits

6. (ab:("community group" OR "community groups" OR "community program" OR "community programs" OR "community programme" OR "community programmes" OR "community intervention" OR "community interventions" OR "community rehabilitation" OR "community mobilisation" OR "community mobilization" OR "community club" OR "community clubs" )) AND (tw:(economic* OR cost* OR price OR prices OR pricing OR pharmacoeconomic* OR expenditure* OR "value for money" OR budget* OR "return on investment" OR roi OR sroi)) 2014-2021 - 28 hits

7. ((ti:(microfinanc* OR micro-financ* OR microcredit OR micro-credit* OR microsaving* OR micro-saving* OR microloan* OR micro-loan* OR microinsurance OR micro-insurance OR microenterpris* OR micro-enterpris* OR "income generating" OR "income generation" OR "loan group" OR "loan groups" OR "livelihood group" OR "livelihood groups")) AND (tw:(economic* OR cost* OR price OR prices OR pricing OR pharmacoeconomic* OR expenditure* OR "value for money" OR budget* OR "return on investment" OR roi OR sroi)) 2014-2021 - 11 hits

8. (ab:(microfinanc* OR micro-financ* OR microcredit OR micro-credit* OR microsaving* OR micro-saving* OR microloan* OR micro-loan* OR microinsurance OR micro-insurance OR microenterpris* OR micro-enterpris* OR "income generating" OR "income generation" OR "loan group" OR "loan groups" OR "livelihood group" OR "livelihood groups")) AND (tw:(economic* OR cost* OR price OR prices OR pricing OR pharmacoeconomic* OR expenditure* OR "value for money" OR budget* OR "return on investment" OR roi OR sroi)) 2014-2021 - 37 hits

9. (mh:("Financing, Organized" OR "Financial Support" )) AND (tw:(economic* OR cost* OR price OR prices OR pricing OR pharmacoeconomic* OR expenditure* OR "value for money" OR budget* OR "return on investment" OR roi OR sroi)) 2014-2021 - 32 hits

**References**

1. Cochrane Effective Practice and Organisation of Care group. LMIC filters. The Cochrane Collaboration. 2020 [accessed 28^th^ April 2021]. Available from: <https://epoc.cochrane.org/lmic-filters>

2. Centre for Reviews and Dissemination. Search strategies for NHS EED. [accessed 28^th^ April 2021]. Available from: <https://www.crd.york.ac.uk/crdweb/searchstrategies.asp>
